# Acrolein-stressed threshold adaptation alters the molecular and metabolic bases of an engineered *Saccharomyces cerevisiae* to improve glutathione production

**DOI:** 10.1038/s41598-018-22836-2

**Published:** 2018-03-14

**Authors:** Wenlong Zhou, Yan Yang, Liang Tang, Kai Cheng, Changkun Li, Huimin Wang, Minzhi Liu, Wei Wang

**Affiliations:** 1 State Key Laboratory of Bioactive Substance and Function of Natural Medicines, Institute of Materia Medica, Peking Union Medical College & Chinese Academy of Medical Sciences, 1 Xian Nong Tan St., 100050 Beijing, China; 2 Key Laboratory of Biosynthesis of Natural Products of National Health and Family Planning Commission, Institute of Materia Medica, Peking Union Medical College & Chinese Academy of Medical Sciences, 1 Xian Nong Tan St., 100050 Beijing, China; 3 College of Life Science, Qufu Normal University, 273165 Qufu, Shandong China; 4 Shimadzu (China) Co., Ltd. Beijing Branch, Chaoyangmen Wai St., 100020 Beijing, China

## Abstract

Acrolein (Acr) was used as a selection agent to improve the glutathione (GSH) overproduction of the prototrophic strain W303-1b/FGP^PT^. After two rounds of adaptive laboratory evolution (ALE), an unexpected result was obtained wherein identical GSH production was observed in the selected isolates. Then, a threshold selection mechanism of Acr-stressed adaption was clarified based on the formation of an Acr-GSH adduct, and a diffusion coefficient (0.36 ± 0.02 μmol·min^−1^·OD_600_^−1^) was calculated. Metabolomic analysis was carried out to reveal the molecular bases that triggered GSH overproduction. The results indicated that all three precursors (glutamic acid (Glu), glycine (Gly) and cysteine (Cys)) needed for GSH synthesis were at a relativity higher concentration in the evolved strain and that the accumulation of homocysteine (Hcy) and cystathionine might promote Cys synthesis and then improve GSH production. In addition to GSH and Cys, it was observed that other non-protein thiols and molecules related to ATP generation were at obviously different levels. To divert the accumulated thiols to GSH biosynthesis, combinatorial strategies, including deletion of cystathionine β-lyase (STR3), overexpression of cystathionine γ-lyase (CYS3) and cystathionine β-synthase (CYS4), and reduction of the unfolded protein response (UPR) through up-regulation of protein disulphide isomerase (PDI), were also investigated.

## Introduction

Glutathione (γ-L-glutamyl-L-cysteinyl-glycine, GSH), which is synthesized from glutamic acid (Glu), cysteine (Cys) and glycine (Gly), is the most abundant non-protein thiol compound in almost all organisms. Its unique structure of a γ-carboxyl of glutamate and a free sulfhydryl moiety of the Cys residue give this tripeptide a wide variety of biological activities, such as anti-oxidation^1^, detoxification^2,3^ and immune regulation^4^. GSH plays a pivotal role in maintaining an appropriate redox environment for organisms and is used as a supplement in various pharmaceuticals^2^.

To date, yeast fermentation is the most common method of GSH production. However, GSH biosynthesis is strictly controlled by a complex regulatory system involving several factors, including the feedback inhibition of γ-glutamylcysteine synthetase (GSH1), substrate limitation and the intracellular redox state. Much effort has been expended to obtain a GSH overproducing strain using the strategies of metabolic engineering, which mainly focus on improving the capacity of the GSH biosynthetic pathway^5^, reducing GSH degradation^6^, promoting GSH secretion^7^, engineering the sulphate assimilation pathway^8^, and increasing the efficiency of ATP utilization^9^. However, the GSH content of the obtained strains only ranges from 1–2%. The regulatory complexity inside the cell restricts the ability to rationally engineer it.

Acrolein (Acr), the most reactive α, β-unsaturated aldehyde, is a ubiquitous environmental pollutant, and its toxicity pertaining to human diseases occurs primarily via protein and DNA adduction resulting in cellular dysfunction. GSH, a native scavenger, has been demonstrated to play a prime role in the cellular defence against Acr^10^. This observation indicates that Acr has a tight relationship with GSH. Moreover, Acr has been used for the selection of GSH overproducing strains^11^. However, the detailed adaptive mechanisms in yeast cells stressed with Acr remained unclear.

In our previous study, a three-pathway engineered stain W303-1b/FGP was constructed. However, the regulation of GSH biosynthesis is far more than pathway engineering, as the GSH level is strictly controlled by a complex regulatory system. To further improve the GSH production of the engineered strain, an adaptive laboratory evolution (ALE) experiment was carried out. Subsequently, a threshold selection mechanism was clarified, and a metabolomic analysis of the evolved strain was performed to elucidate the augmented thiol compounds involved in the enhancement of GSH levels, guiding the re-engineering of the GSH biosynthetic pathway.

## Results and Discussion

### ALE of the engineered strain

To ensure that the engineered strain W303-1b/FGP could be employed in WMVIII medium, its five auxotrophic genes were reversed using the CRISPR/Cas9-mediated gene editing method^12,13^, generating the prototrophic W303-1b/FGP^PT^ strain (see supplementary file). The strains W303-1b/FGP and W303-1b/FGP^PT^ presented approximately 216 mg/L GSH (Supplementary Fig. S1A) and a maximum DCW of approximately 9.3 g/L in YPD medium (Supplementary Fig. S1B). The results showed that the prototrophic strain has the same capacity for glutathione production as the auxotrophic strain and was more suitable for evolution. Prior to undertaking the ALE experiments, the stability of GSH production of the prototrophic strain W303-1b/FGP^PT^ and W303-1b/FGP^PT^m (W303-1b/FGP^PT^ pretreated with 50 μg/mL MNNG) was examined to ensure that the changes in GSH levels stemmed from evolution. The GSH levels in both strains cultured in WMVIII medium reached 108 mg/L. This result indicated that the GSH production of both strains changed little after 100 generations (Supplementary Fig. S2A,B).

**Figure 1:**
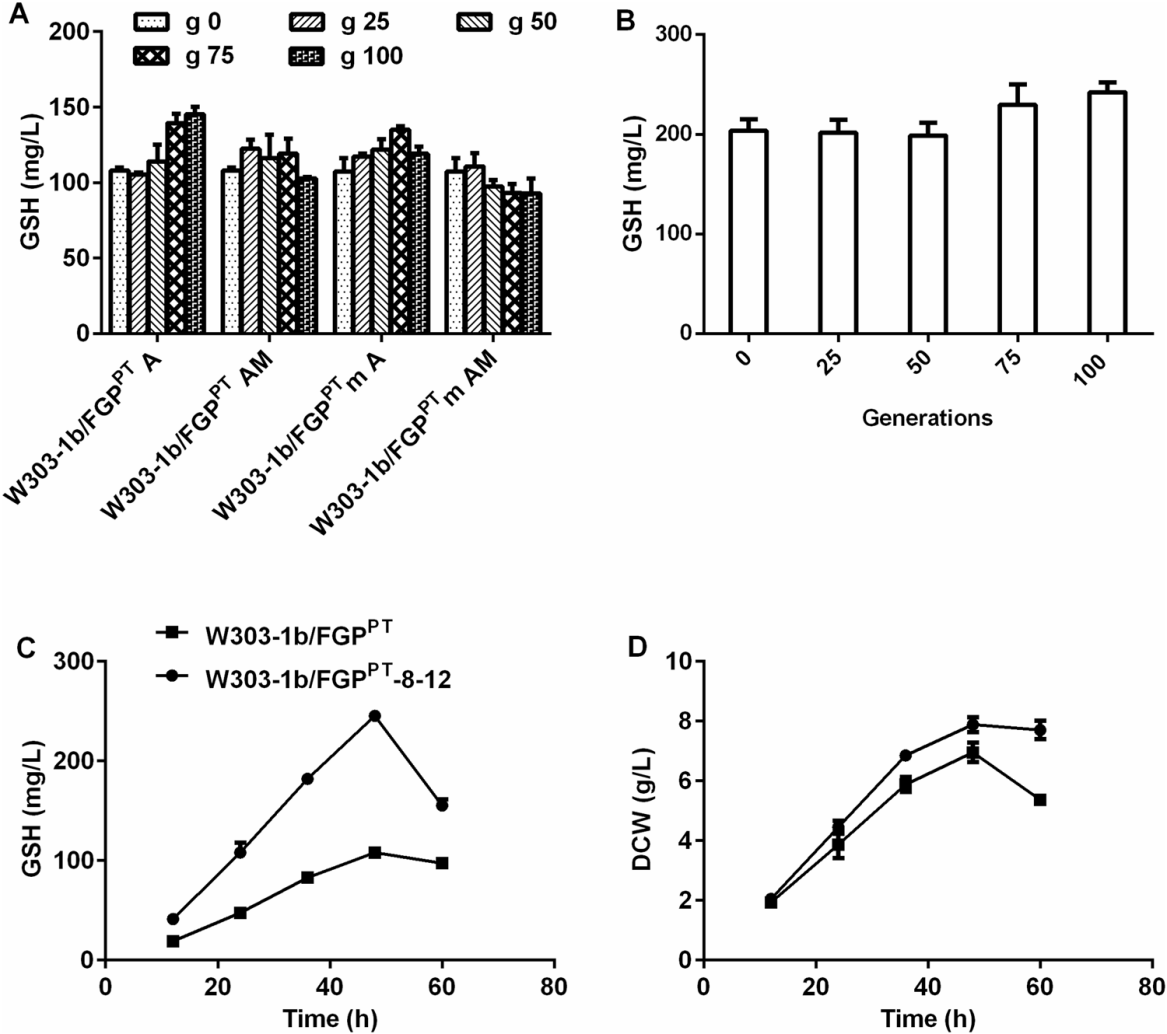
Progress of the selection of GSH-overproducing strains in shaken flasks. (**A**) Glutathione was measured at the indicated generation times after cultivation for 48 h in WMVIII media. The selection started from strains W303-1b/FGP^PT^ and W303-1b/FGP^PT^m. The population was grown in WMVIII medium supplemented initially with 0.2 mM Acr and exposed to sequential serial passages by increasing the Acr dose. (**B**) The second round of the ALE experiments started from the isolate W303-1b/FGP^PT^-8 after the first-round ALE experiment. Glutathione was measured as described above. (**C**) GSH accumulation of the parental strain W303-1b/FGP^PT^ and the evolved strain W303-1b/FGP^PT^-8–12. (**D**) Growth behaviour of the parental strain and the evolved strain. ‘m’ refers to yeast strains treated with 50 μg/mL MNNG before the ALE experiment was conducted. ‘A’ and ‘AM’ refer to the ALE assay using Acr as a selection agent without or with MNNG. The values are presented as the means, and the error bars show the SD (n = 3). When not shown, error bars lie within the symbols.

Usually, continuous addition of MNNG, a chemical mutagenic strategy to increase the genetic diversity of the strains, might be more suitable to shorten the adaption period^14^. First, 5 μg/mL MNNG was added during adaption, and an initial concentration of 0.2 mM Acr inhibiting the growth of both strains was chosen to initiate the adaptation assay (Supplementary Table S1). Four populations (W303-1b/FGP^PT^ AM, W303-1b/FGP^PT^m AM, W303-1b/FGP^PT^ A and W303-1b/FGP^PT^m A (‘AM’ and ‘A’ refer to the ALE assay using Acr as a selection agent with or without addition of MNNG)) were monitored by measuring their GSH amounts at continuous intervals. After approximately 100 generations, the concentration of Acr had increased to 0.4 mM. The pools of W303-1b/FGP^PT^ A-100 and W303-1b/FGP^PT^m A-75 had GSH levels of 145.3 ± 4.8 mg/L and 135.3 ± 2.2 mg/L (Fig. 1A), respectively. Although the presence of MNNG can markedly increase the rate of mutation, it appears that the efficiency of ALE was not associated with the genetic diversity of the parental strain and that neither the MNNG treatment in a single high dosage nor a continuous addition of the mutagen contributed to GSH accumulation. It may be that mutagenesis can emerge naturally during the process of ALE, while a higher mutation rate to a certain extent, can also result in beneficial genetic load.

Twenty single colonies from each population were cultivated in liquid WMVIII medium. Only three colonies from W303-1b/FGP^PT^ A-100 showed significantly higher GSH production than their parental strain, while a consistent change in GSH production was found in the isolates of W303-1b/FGP^PT^m A-75 (Supplementary Fig. S3A,B).

To select the preponderant strain used for the second round of direct evolution, six isolates were cultured in YPD medium. As shown in Supplementary Fig. S4A, the higher GSH production of the three isolates from W303-1b FGP^PT^m A-75 was 229 ± 4.9 mg/L. W303-1b/FGP^PT^-8 from pool W303-1b/FGP^PT^ A-100 had the highest GSH accumulation, up to 252.3 ± 7.7 mg/L GSH. Although W303-1b/FGP^PT^m-18 had a similar GSH content (3.6 ± 0.2%) compared to W303-1b/FGP^PT^-8, its DCW was the lowest (6.1 ± 0.2 g/L) of the six (Supplementary Fig. S4B). As a result, W303-1b/FGP^PT^-8 with stable GSH production (Supplementary Fig. S2C) was selected for the second round of evolution.

A second evolutionary experiment was carried out using the best-evolved isolate, W303-1b/FGP^PT^-8. GSH production of the evolved strain was measured at specified intervals (Fig. 1B). After 100 generations, the GSH production of the evolved pool reached 242.3 ± 10.1 mg/L, and the corresponding Acr doses increased to 1.24 mM. Subsequently, the GSH production of forty single isolates from this population was analysed. Surprisingly, the amount of GSH in the isolates revealed almost identical GSH production as the pool with a similar biomass. Finally, isolate W303-1b/FGP^PT^-8–12, with a maximum GSH production of 245.4 ± 5.2 mg/L at 48 h, was selected as the final evolved strain due to a relatively higher biomass of 7.0 ± 0.3 g/L (Fig. 1C,D). Its stability of GSH production is shown in Supplementary Fig. S2D.

In summary, after two rounds of adaptive evolution, the GSH-overproducing strain W303-1b/FGP^PT^-8–12 was obtained. It should be noted that in the second round of ALE, the evolved cells that grew in Acr-containing medium for more than 100 generations did tolerate higher concentrations of Acr, and no higher GSH-accumulating strains were obtained. Based on these results, it can be hypothesized that there is a diffusion threshold of Acr across the membrane or a neutralization mechanism that attenuates cellular toxicity and selection stress of Acr to improve the GSH production of the evolved populations.

### Acr is a threshold selection agent for GSH overproduction in *S. cerevisiae*

After the second round of selection, we obtained the unexpected result that forty isolates had an almost identical GSH titre. What failed to produce the selection pressure? This inspired us to explore a problematic molecular mechanism eliminating the beneficial fitness. To date, there is only one literature report that Acr might cross the cell membrane by means of aquaglyceroporin-mediated passive diffusion^15^. We therefore supposed that there was a limit on the formation of a selection pressure during the process of Acr transmembrane transport in the evolved strain, while the endogenous GSH rapidly detoxified Acr to form the less-toxic Acr-GSH adduct.

To test this hypothesis, a toleration assay against much higher concentrations of Acr was carried out to test cell survival in the evolved strain W303-1b/FGP^PT^-8–12. Although treatment with 1.6, 1.8, or 2.0 mM Acr greatly exceeded the final 1.24 mM of adaptation, the survival rates of W303-1b/FGP^PT^-8–12 cell sets showed much less difference with a *P* value > 0.05 (Supplementary Table S2). A second finding was that during the second round of selection, it took the passage population of each transfer 24 h less than the first-round selection to grow from an initial OD_600_ of 0.5 to an early mid-exponential phase (OD_600_ of ~15) (data not shown), which could be because the GSH production capacity of the evolved strain nearly counteracted the stressed pressure of Acr of each transfer, leading to a lack of severe growth inhibition. Another reason was that, although a concentration gradient between extracellular and intracellular Acr formed, the passive diffusion of Acr across the cell membrane might heavily rely on the transport capacity of aquaglyceroporin FPS1, the loss of which confers resistance to Acr in *S. cerevisiae*^15^.

**Figure 2:**
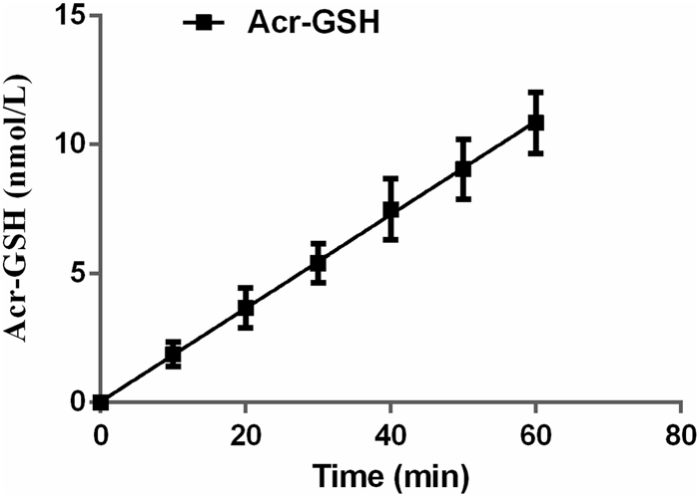
The accumulation of Acr-GSH in the evolved strain W303-1b/FGP^PT^-8–12 in the first 60 min of treatment with 1.2 mM Acr. The equation for the linear region was y = 0.18 (±0.01) x + 0.04 (±0.30) with a correlation coefficient (*R*^*2*^) of 0.961.

Subsequently, the constant diffusion rate along with the stable accumulation of the Acr-GSH adduct was determined by LC-MS/MS using the prepared Acr-GSH as a reference standard (Supplementary Table S4, Figs S5 and S9D) because not only is Acr very active and volatile, but also, GSH, the most abundant thiol in the evolved strains, can quickly react with Acr to form the stable Acr-GSH adduct. The cultured mid-exponential growth phase strain W303–1b/FGP^PT^-8–12 was treated with 1.2 mM Acr in WMVIII medium after dilution to an OD_600_ of 0.5. The conjugative reaction ratio between Acr and GSH was 1:1. The accumulation of Acr-GSH up to 12.5 nmol/L was more than the depletion of GSH (5.0 nmol/L), indicating that the process of GSH biosynthesis was constant. Moreover, it should be noted that in a single treatment with a higher dose of Acr, the accumulation of Acr-GSH in the first 60 min was nearly linear (Fig. 2). A stable diffusion coefficient of 0.36 ± 0.02 μmol·min^−1^·OD_600_^−1^ was calculated based on Acr-GSH accumulation. However, GSH depleted rapidly after exposure to Acr and remained at 0.3 nmol/L after 90 min. When the culture time extended, the intracellular GSH levels increased, as observed during the process of ALE. Different from GSH, the accumulation of Acr-GSH continued to increase over 90 min. After 90 min, the Acr-GSH concentration steeply declined (Supplementary Fig. S6). This was attributed to the induction of the expression of orthologous homologs of aldehyde dehydrogenase (ALDH) and alcohol dehydrogenase (ADH), or old yellow enzyme (OYE2), which might contribute to the liner region change of Acr-GSH accumulation occurring in the treated cells. Thus, the Acr-GSH adduct can accumulate before the enzyme systems significantly mediate the oxidation or reduction pathways of metabolic degradation^16–18^. Although during the second round of selection, a higher concentration gradient between extracellular and intracellular Acr can occur, cellular homeostasis between the permeated Acr and biosynthesized thiols (e.g., GSH) forms an identical selection trait, leading to no beneficial phenotype or fitness to improve GSH production. Therefore, it is concluded that Acr is a threshold selection agent for GSH production in ALE.

In addition to GSH, Acr can conjugate other endogenous thiols, such as Cys, NAC and Hcy, which were obviously increased compared to the parental strain W303-1b/FGP^PT^, as described below. The determination of Acr-GSH accumulation did not include the minor adducts Acr-Cys, Acr-Hcy and Acr-NAC (Supplementary Fig. S9A–C). Thus, the correlation coefficient (*R*^2^) corresponding to the narrow linear region of Acr-GSH formation was 0.961.

### Comparative metabolomic analysis of the evolved strain

**Figure 3:**
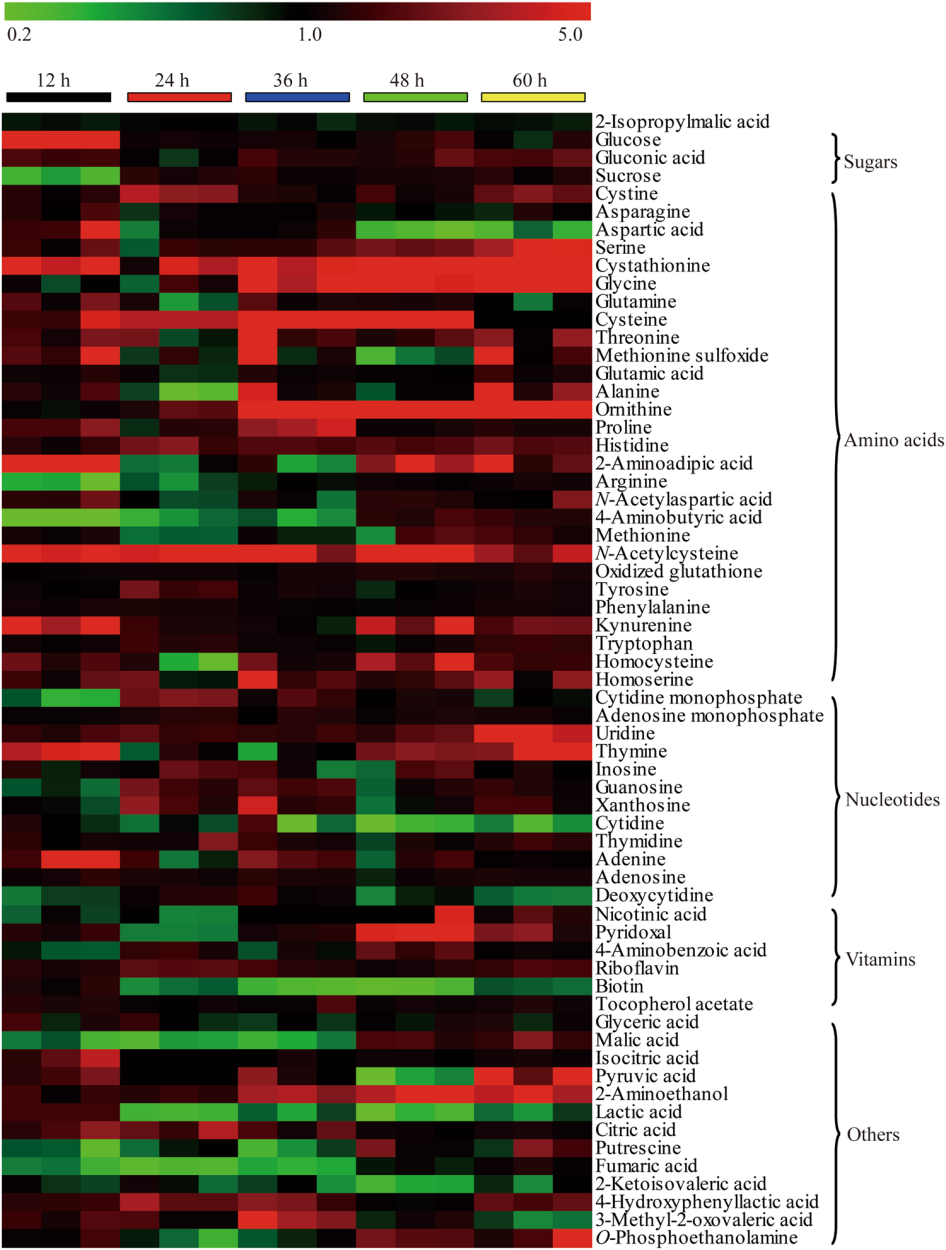
Heat map of the metabolites from the evolved strain W303-1b/FGP^PT^-8–12 obtained from the ALE experiments and the parental strain W303-1b/FGP^PT^. Rows represent specific intracellular metabolites. Columns represent different cell growth intervals (12, 24, 36, 48 and 60 h). Fold change was used to evaluate the relative concentration of metabolites of W303-1b/FGP^PT^-8–12 and W303-1b/FGP^PT^.

According to the results of the ALE experiments, there was a question as to what enabled the evolved strain to accumulate higher intracellular GSH levels. To better understand the molecular and metabolic bases of GSH overproduction, a comparative metabolite analysis between the parental strain and the evolved strain was undertaken, and 62 comparable metabolites (Supplementary Table S3) were identified. As shown in the heat map in Fig. 3, the significant differences comprised 27 amino acids, 11 organic acids, 3 sugars, 5 vitamins, 12 nucleotides, and 4 miscellaneous compounds.

Among the comparable metabolites, most amino acids were at significantly higher levels in the evolved strain W303-1b/FGP^PT^-8–12. In particular, the relative concentration ratios of Cys and Gly were more than 11- and 5-fold increased, respectively (Supplementary Table S3 and Fig. S7). The concentration of Glu was also slightly higher in the evolved strain. It might be that Glu itself remained at a high concentration during the fermentation process^19^. It was noted that the levels of intermediates, such as L-aspartate, homoserine, Hcy and cystathionine, in the Cys biosynthetic pathway varied greatly. The ratios of these intermediates at 48 h, when the accumulation of GSH reached its maximum, were on average 0.3, 2.0, 3.9 and 10.3, respectively (Supplementary Table S3 and Fig. S7). The continuous declining ratio of L-aspartate from 2.2 at 12 h to 0.3 at 48 h accompanied by the increase in GSH accumulation indicated that the evolved W303-1b/FGP^PT^-8–12 had a higher capacity of sulphur assimilation. That is, the accumulated homoserine converted to Hcy synthesis. Consequently, Hcy and cystathionine contributed to a higher Cys accumulation and further led to an increased GSH production. Unexpectedly, we detected the strongest changes in ornithine, along with some less pronounced changes in arginine, for which we could not identify their physiological functions in the evolved strain.

In addition to the increased intermediates of GSH production, adequate ATP supplementation also triggers GSH overproduction in the evolved strain. In the engineered W303-1b/FGP^PT^-8–12 cells, the ratios of glyceric acid, isocitric acid, pyruvic acid and citric acid, involved in glycolysis and the citrate cycle, were also >1 (Supplementary Fig. S7). This indicated that more ATP regeneration promoted consecutive ATP-dependent GSH synthesis in the evolved strain compared to the parent strain, resulting in the enhancement of GSH productivity^20^.

In addition to the two concerns mentioned above, the evolved strain also presented a much ‘stronger’ metabolic capacity, as follows: the higher accumulation of the four nucleotides along with their intermediates would promote cellular DNA synthesis, and the enhancements in the vitamins nicotinic acid, pyridoxal, riboflavin and tocopherol acetate might improve the catalytic efficiencies of metabolic enzymes. The increase in *O*-phosphoethanolamine and 2-aminoethanol might promote phospholipid biosynthesis. Thus, these metabolic alternations indicate that the resulting strain has evolved to a multivariate fitness, and the corresponding metabolic processes may be distributed among most of the major biological processes^18^.

### Identification of active thiols and detoxification mechanisms against Acr

In addition to the enhanced accumulation of GSH and precursor Cys along with its biosynthetic intermediates (e.g., Hcy and cystathionine), it was clearly observed that the amount of NAC, a powerful antioxidant amino acid, was heavily increased by 10-fold at 48 h compared to the parental strain (Fig. 3). A supplemental assay result showed that the different dosages of NAC added to the cultured W303-1b/FGP^PT^ did not promote GSH production (data not shown). Consequently, we supposed that all the thiols enhanced in the adaption process might be directly involved in the detoxification of Acr through the formation of less-toxic Acr-thiols^21^. To determine whether other non-protein thiols besides GSH directly reacted with Acr *in vivo*, two independent Acr-stress experiments were carried out. After the W303-1b/FGP^PT^ strain grew for 36 h (OD_600_ = ~52), a set of 30-mL cultures were removed, and then, sub-lethal and lethal dosages of Acr were directly added into two aliquots of culture and incubated for an additional 1 h or 6 h. Even after the conjugation reaction of 1 h, the GSH and Cys in the cells treated with two different incubations could not be detected, as shown in Supplementary Fig. S8. To further confirm these results, another two Acr-Hcy and Acr-NAC samples were monitored by LC-MS analysis using the corresponding Acr-thiol adducts as the reference standards prepared by *in vitro* chemical reactions (Supplementary Fig. S9A–C). All the thiols were almost depleted and formed the corresponding Acr-thiol adducts (Supplementary Fig. S10A–D). Hence, it can be proposed that, in the Acr-stress evolution, a forward selective pressure enables the sulphur flux of the evolved strain to be diverted to produce more thiols, directly neutralizing the intracellular toxicity of Acr. Once the Acr-stress selection is removed, the positively selected strain still inherits a stronger capacity for sulphur assimilation and accumulates more NAC along with other thiol intermediates, including Hcy, Cys and cystathionine, which further contribute to GSH production. Thus, based on the present work and previous observations^22^, it is concluded that the action mechanism of the stress-induced thiols is to directly detoxify Acr upon exposure to the cells.

### Regulation of the Cys biosynthetic pathway

**Figure 4:**
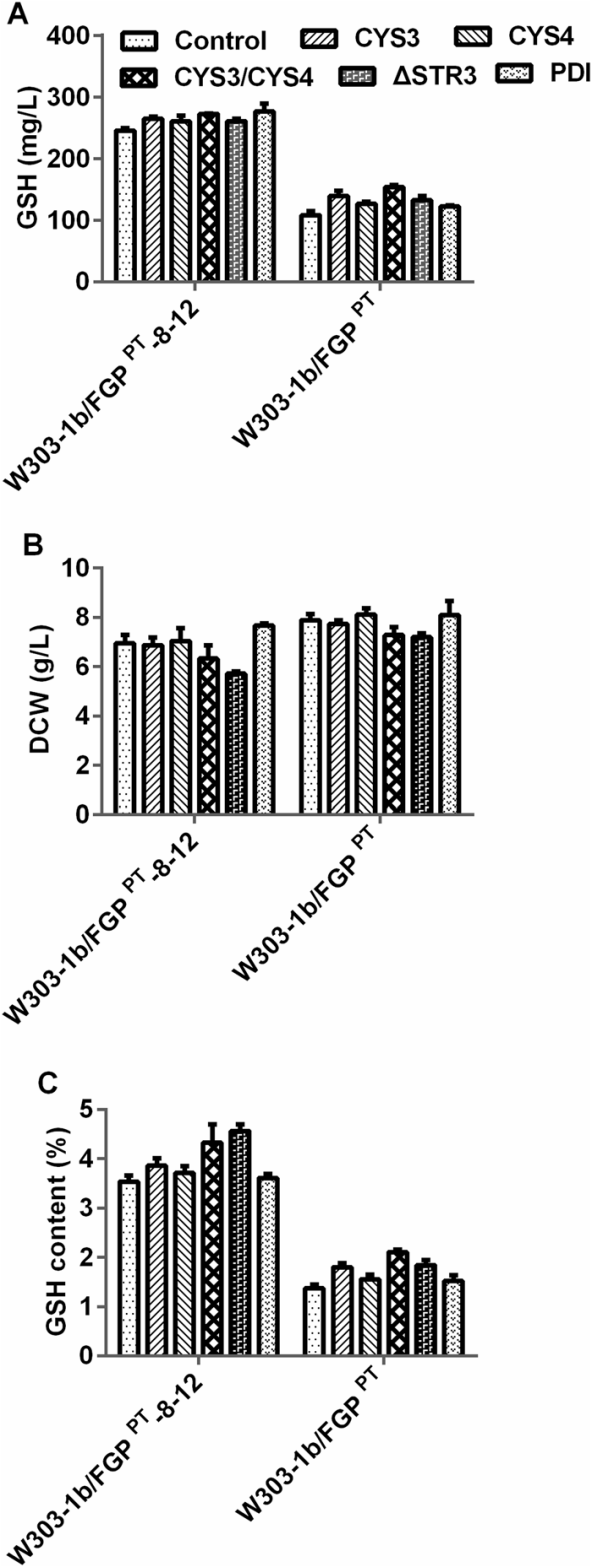
The effects of modulation of the Cys synthesis pathway and overexpression of PDI on the GSH accumulation and DCWs of the evolved strain W303-1b/FGP^PT^-8–12 and the engineered strain W303-1b/FGP^PT^. (**A**) GSH production (mg/L). (**B**) DCW (g/L). (**C**) GSH content (%). The values are presented as the means, and the error bars show the SD (n = 3).

Cys has been shown to be the major limiting precursor for GSH accumulation. GSH is synthesized from the amino acids Glu, Cys and Gly. The extra addition of Cys could greatly increase the GSH content at the special fermentation stage^23,24^. It was assumed that the use of a stable supply of intracellular Cys, via the modulation of the Cys biosynthetic pathway, would be a promising strategy for GSH production. To test whether it was feasible to divert the Cys precursors (i.e., Hcy and cystathionine) accumulated in the evolved strain to Cys synthesis and then increase GSH production, the two key genes encoding cystathionine γ-lyase (γ-CTLase, *CYS3*) and cystathionine β-synthase (β-CTSase, *CYS4*) of the Cys biosynthesis pathway were independently or simultaneously overexpressed in the evolved strain under the control of a constitutive promoter (Supplementary Fig. S11A,B). The GSH production of the engineered strains W303-1b/FGP^PT^-8–12/CYS3, W303-1b/FGP^PT^-8–12/CYS4 and W303-1b/FGP^PT^-8–12/CYS3/CYS4 increased to 264 mg/L, 260 mg/L and 272 mg/L, respectively (Fig. 4A). The results indicated that up-regulation of the Cys biosynthetic pathway might change the flux to Cys and improve GSH production. The DCWs of the two strains W303-1b/FGP^PT^-8–12/CYS3 and W303-1b/FGP^PT^-8–12/CYS4 were the same as that of the control (7.0 ± 0.3 g/L). However, the DCW of the co-overexpressed strain W303-1b/FGP^PT^-8–12/CYS3/CYS4 decreased to 6.3 ± 0.5 g/L (Fig. 4B). This slight defect in growth inhibition might result from the overexpression of both CYS3 and CYS4, which will be discussed in the next section. These findings were in agreement with those for engineered W303-1b/FGP^PT^/CYS3, W303-1b/FGP^PT^/CYS4 and W303-1b/FGP^PT^/CYS3/CYS4, which increased 139.3 ± 8.2 mg/L, 126.3 ± 3.4 mg/L and 153.4 ± 3.5 mg/L, respectively (Fig. 4A). In earlier studies, researchers had overexpressed CYS4 and CYS3 in *S. cerevisiae* DBY7286, which had less effect on GSH production^9^. This conflicting result might be due to the poor Cys precursors for utilization and conversion to GSH compared to those of the evolved strain. These results assume that the overexpression of genes involved in the Cys biosynthetic pathway has the potential to improve GSH production. In addition to the strategy described above, Cys production can be enhanced by disrupting the Cys degradation genes^6^ or by metabolic engineering of sulphur metabolism^8^. Combining the results of this work, it is evident that Cys overproduction depends on stronger sulphur assimilation, i.e., the accumulation of the intermediates Hcy and cystathionine boosting GSH production.

### Deletion of *STR3* to improve GSH synthesis

In *S. cerevisiae*, Hcy is the first sulphur amino acid to be generated by the condensation of both sulphide and *O*-acetylhomoserine, and Cys is exclusively synthesized from Hcy via intermediate cystathionine^25,26^. Cys and Hcy can be interconverted in forward and reverse trans-sulphuration pathways, in which STR3 reversely cleaves cystathionine via an α,β-elimination reaction to produce Hcy and the by-products pyruvate and ammonia^27^ and maintain the physiological cycle between cystathionine and Hcy.

Because marked levels of both Hcy and cystathionine accumulated in the evolved strain (Fig. 3), we assumed that the cycle between Hcy and cystathionine could be broken and the sulphur flux redirected to further improve cystathionine synthesis and enhance GSH production. To examine this hypothesis, we constructed *ΔSTR3* strains of both W303-1b/FGP^PT^ and W303-1b/FGP^PT^-8–12 to determine the effects of the disruption of *STR3* on the accumulation of GSH (Supplementary Fig. S12). The mutants W303-1b/FGP^PT^/*ΔSTR3* and W303-1b/FGP^PT^-8–12/*ΔSTR3* produced higher GSH levels of 132.5 ± 7.2 mg/L and 261.0 ± 3.8 mg/L, respectively (Fig. 4A), than the parental strains (108.2 ± 6.8 mg/L and 245.4 ± 5.2 mg/L, respectively). It was clear that the inactivation of *STR3* lowered the DCW of W303-1b/FGP^PT^-8–12/*ΔSTR3* with a higher GSH content, while the growth of W303-1b/FGP^PT^/*ΔSTR3* reached almost the same biomass as the parental strain (Fig. 4B,C). This DCW difference indicated that the deletion of STR3 did not significantly lead to the growth defect of the engineered yeast, and the higher accumulation of GSH might result in the slight growth inhibition of W303-1b/FGP^PT^-8–12/*ΔSTR3*. Although previous research showed that yeast cells in the absence of *STR3* were unable to grow on Cys or cystathionine as the sole sulphur source^28^, this culture condition does not occur in the evolved strain. Thus, the gene deletion of *STR3* is a feasible strategy to boost GSH production by redirecting the sulphur flux.

### Overexpression of protein disulphide isomerase (PDI) to raise GSH production

PDI is essential for the formation of disulphide bonds in protein folding^29^. The formed oxidized PDIs will be re-reduced by GSH, which maintains the balance of oxidized and reduced PDIs. Many of the protein folding maturation processes occurring in the endoplasmic reticulum (ER) are regulated by a mechanism referred to as the unfolded protein response (UPR) under conditions of protein misfolding or unfolded stress^30^. In the present study, three GSH biosynthetic pathways were combined to be overexpressed in the engineered strain W303-1b/FGP^PT^. Therefore, there was a question as to whether the up-regulation of these enzymes exceeded the cell’s capacity to properly fold protein, initiating UPR and resulting in a loss of protein expression. To unveil the role of PDI in GSH accumulation, an additional *PDI1* was expressed in both the engineered strain W303-1b/FGP^PT^ and the evolved strain W303-1b/FGP^PT^-8–12 under the control of the phosphoglycerate kinase 1 (PGK1) promotor of *S. cerevisiae* (Supplementary Fig. S11C). Together with the overexpression of *PDI1*, the intracellular GSH yield of the engineered W303-1b/FGP^PT^ increased to 122.2 ± 2.1 mg/L (Fig. 4A). Meanwhile, the GSH production of the evolved W303-1b/FGP^PT^-8–12/PDI reached 276.5 ± 3.0 mg/L, and its DCW increased to 7.7 ± 0.1 g/L (Fig. 4B). When *PDI1* was overexpressed in the wild strain W303-1b, there was no change in either GSH production or DCW (data not shown). Taken together, these results showed that the overexpression of the additional *PDI1* attenuated the growth inhibition induced by the UPR effects of protein misfolding, along with the increased DCW and GSH contents compared to the parental strains.

### Combining modulation of *PDI*, *CYS3* and *CYS4* for GSH production

**Figure 5:**
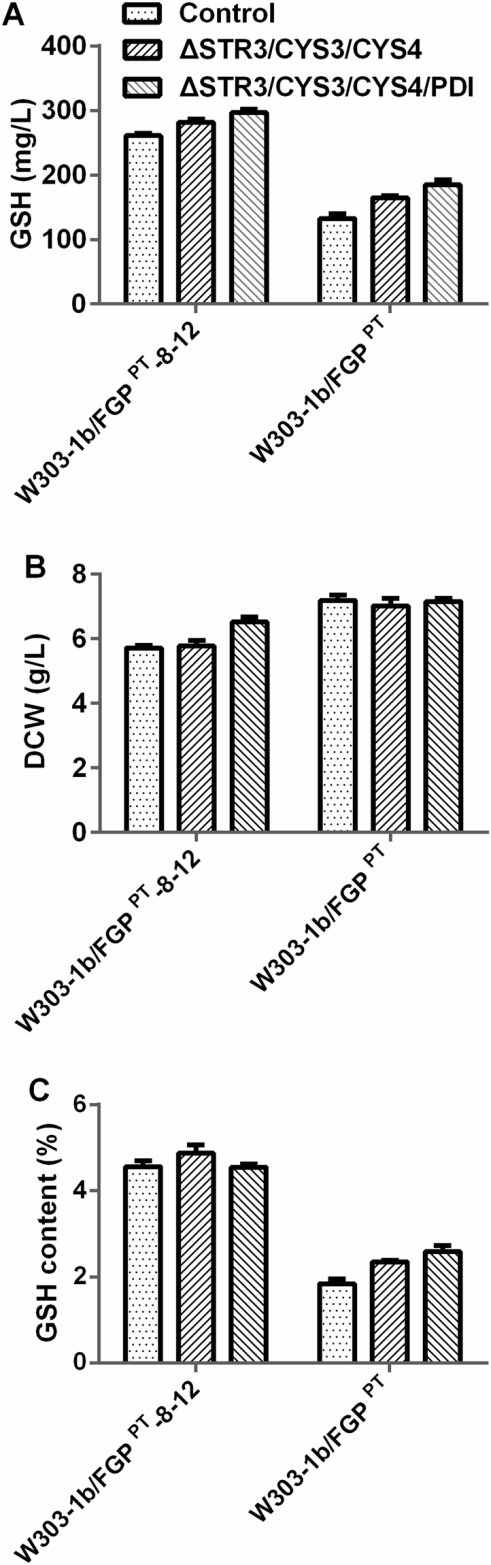
GSH production and DCW of combinatorial strains with the modulation of both the Cys biosynthetic pathway and PDI. (**A**) GSH production of combinatorial strains based on the evolved strain W303-1b/FGP^PT^-8–12 and the engineered strain W303-1b/FGP^PT^. (**B**) DCW (g/L). (**C**) GSH content (%). The values are presented as the means, and the error bars show the SD (n = 3). ‘Control’ represents W303-1b/FGP^PT^-8–12/*ΔSTR3* and W303-1b/FGP^PT^/*ΔSTR3*, respectively.

To obtain a higher GSH concentration, the combinatorial up-regulation of *PDI1*, *CYS3* and *CYS4* was further investigated. Strains W303-1b/FGP^PT^/*ΔSTR3* and W303-1b/FGP^PT^-8–12/*ΔSTR3* with a higher GSH synthetic capacity were chosen as the starting strains for the further introduction of *CYS3*, *CYS4* and *PDI1* via independently or combinatorially integrated linearized genes. The four combinatorial strains W303-1b/FGP^PT^/*ΔSTR3/CYS3*/*CYS4*, W303-1b/FGP^PT^/*ΔSTR3/CYS3*/*CYS4*/*PDI*, W303-1b/FGP^PT^-8–12/*ΔSTR3/CYS3*/*CYS4* and W303-1b/FGP^PT^-8–12/*ΔSTR3/CYS3*/*CYS4*/*PDI* were constructed. We next measured the GSH production of these strains after growth for 48 h in WMVIII medium. The GSH production of W303-1b/FGP^PT^/*ΔSTR3/CYS3*/*CYS4* and W303-1b/FGP^PT^/*ΔSTR3/CYS3*/*CYS4*/*PDI* was improved to 164.7 ± 3.3 mg/L and 184.7 ± 7.9 mg/L, respectively (Fig. 5A), along with the same DCW of approximately 7.0 g/L. As expected in W303-1b/FGP^PT^-8–12, when CYS3 and CYS4 were co-overexpressed in the STR3 mutant W303-1b/FGP^PT^-8–12/*ΔSTR3/CYS3*/*CYS4*, the GSH production increased to 281.6 ± 4.8 mg/L, while the DCW decreased to 5.8 ± 0.2 g/L (Fig. 5B), resulting in a GSH content of 4.5 ± 0.1% (Fig. 5C). To reduce the metabolic stress in the modified strain, *PDI1* was introduced. Accordingly, the W303-1b/FGP^PT^-8–12/*ΔSTR3/CYS3*/*CYS4*/*PDI* strain showed a particularly high GSH production of 296.3 ± 5.6 mg/L, 1.21-fold higher than that of the control strain W303-1b/FGP^PT^-8–12, and its DCW reached 6.5 ± 0.2 g/L, higher than that of W303-1b/FGP^PT^-8–12/*ΔSTR3* and W303-1b/FGP^PT^-8–12/*ΔSTR3*/*CYS3*/*CYS4* (Fig. 5B). It could be inferred that PDI overexpression partially restored the growth inhibition due to the co-expression of CYS3 and CYS4. These results indicated that the deletion of STR3 and overexpression of CYS3, CYS4 and PDI function synergistically to attain dramatically increased intracellular GSH production. However, how the disruption of the STR3 in the engineered strain W303-1b/FGP^PT^-8–12/*ΔSTR3*/*CYS3*/*CYS4/PDI* affects sulphur assimilation and represses growth needs to be further studied.

The protein misfolding or aggregation that occurs when multiple genes are overexpressed in the host cells usually leads to multiple organelle UPR effects, inhibiting the host growth or inducing cell apoptosis^31,32^. In these physiological processes, PDI expression plays a vital role of assisting the disulphide formation of the proteins expressed, reducing the misfolding protein aggregation and then removing the inhibitory UPR action^33^. In this work, the additional expression of *PDI1* not only improved the GSH accumulation of the engineered W303-1b/FGP^PT^/PDI1 and W303-1b/FGP^PT^-8–12/PDI1 but also boosted the GSH production of W303-1b/FGP^PT^-8–12/*ΔSTR3*/*CYS3*/*CYS4/PDI*, in which the obvious growth inhibition was alleviated compared to W303-1b/FGP^PT^-8–12/*ΔSTR3*/*CYS3*/*CYS4*. The co-overexpression of CYS3 and CYS4 in strain W303-1b/FGP^PT^-8–12/*CYS3*/*CYS4* was observed to cause a stronger growth inhibition than the individual overexpression of CYS3 or CYS4. It is evident that the overexpression of multiple proteins can saturate the cell’s capacity to properly fold proteins, and expressing the additional PDIs will compensate for defects in disulphide formation and isomerization, resulting in an enhanced capacity of the engineered yeast cells for GSH production. Before this, although many efforts focusing on the modulation of the GSH biosynthetic pathway have been made to develop engineered yeasts for GSH production^5^, very little attention has been paid to the fundamental cellular processes, such as the folding of proteins. In this work, the positive regulation of additional PDIs indicates that a complex metabolic process, especially in the synthetic biology industry, requires systemic manipulation to counteract the UPR effects induced by the overexpressed misfolding proteins.

## Conclusions

The adaption mechanism of Acr was elucidated as depending on aquaglyceroporin-mediated constant diffusion. The insights gained from the metabolomic analysis indicated that the accumulation of GSH and other thiols (e.g., Cys and NAC) directly neutralized free Acr. After removing the Acr stress, the improved traits devoted to GSH overproduction along with higher accumulations of Cys and its precursors Hcy and cystathionine underlay the further improvements in GSH synthesis by metabolic engineering. In summary, combining ALE and genetic engineering is a constructive approach to further boost GSH production in *S. cerevisiae*.

## Materials and Methods

### Strains and media

The strains used in this study and their relevant properties are listed in Table 1. *E. coli* Trans1-T1 (TransGen, Beijing, China) was used for the propagation and manipulation of the recombinant DNA. The strain W303-1b/FGP (*MATα ade*2*-1 leu2-3*,*112 his3-11*,*15 ura3-1 trp1-1*) was used as the parental strain. WMVIII medium^11^ and YPD medium (yeast extract 10 g/L, peptone 20 g/L and glucose 20 g/L) were used to culture yeast cells.

**Table 1:** Strains and plasmids used in this study.

Strains and plasmids	Relevant properties	Source or references
**Strains**		
*E. coli* Trans1-T1	*F* ^*-*^ *φ80(lacZ) ΔM15 ΔlacX74 hsdR(rK* ^*−*^ *mK* ^+^ *) ΔrecA1398 endA1 tonA*	Our lab
*S. cerevisiae* W303-1b	*MATα ade2-1 leu2-3,112 his 3-11,15 ura 3-1trp1-1*	Our lab
W303-1b/FGP	The parental strain with the three-pathway engineered strain derived from W303-1b	Tang *et al*., 2015
W303-1b/FGP^PT^	Prototrophic strain, a derivative of W303-1b/FGP via the reversion	This study
W303-1b/FGP^PT^-8	GSH overproduction strain obtained from the first round of ALE experiments	This study
W303-1b/FGP^PT^-8–12	GSH overproduction strain obtained from the second round of ALE experiments	This study
W303-1b/FGP^PT^/CYS3	W303-1b/FGP^PT^ derivative with the integration of pδGAPh-CYS3	This study
W303-1b/FGP^PT^/CYS4	W303-1b/FGP^PT^ derivative with the integration of pδGAPg-CYS4	This study
W303-1b/FGP^PT^/CYS3/CYS4	W303-1b/FGP^PT^ derivative with the integration of pδGAPh-CYS3 and pδGAPg-CYS4	This study
W303-1b/FGP^PT^/ΔSTR3	W303-1b/FGP^PT^ derivative with the disruption of *STR3* gene	This study
W303-1b/FGP^PT^/PDI	W303-1b/FGP^PT^ derivative with the integration pδPGKb-PDI	This study
W303-1b/FGP^PT^/ΔSTR3/CYS3/CYS4	W303-1b/FGP^PT^/ΔSTR3 derivative with the integration of pδGAPh-CYS3 and pδGAPg-CYS4	This study
W303-1b/FGP^PT^/ΔSTR3/CYS3/CYS4/PDI	W303-1b/FGP^PT^/ΔSTR3/CYS3/CYS4 derivative with the integration of pδPGKb-PDI	This study
W303-1b/FGP^PT^-8–12/CYS3	W303-1b/FGP^PT^-8–12 derivative with the integration of pδGAPh-CYS3	This study
W303-1b/FGP^PT^-8–12/CYS4	W303-1b/FGP^PT^-8–12 derivative with the integration of pδGAPg-CYS4	This study
W303-1b/FGP^PT^-8–12/CYS3/CYS4	W303-1b/FGP^PT^-8–12 derivative with the integration of pδGAPh-CYS3 and pδGAPg-CYS4	This study
W303-1b/FGP^PT^-8–12/ΔSTR3	W303-1b/FGP^PT^-8–12 derivative with the disruption of *STR3* gene	This study
W303-1b/FGP^PT^-8–12/PDI	W303-1b/FGP^PT^-8–12 derivative with the integration of pδPGKb-PDI	This study
W303-1b/FGP^PT^-8–12/ΔSTR3/CYS3/CYS4	W303-1b/FGP^PT^-8–12/ΔSTR3 derivative with the integration of pδGAPh-CYS3 and pδGAPg-CYS4	This study
W303-1b/FGP^PT^-8–12/ΔSTR3/CYS3/CYS4/PDI Plasmids	W303-1b/FGP^PT^-8–12/ΔSTR3/CYS3/CYS4 derivative with the integration of pδPGKb-PDI	This study
pδGAPh	pBluescript II KS(+) derivative with homologous δ region, P_gap_, T_pgk1_, HygB^r^	Our lab
pδGAPg	pBluescript II KS(+) derivative with homologous δ region, P_gap_, T_pgk1_, G418^r^	Our lab
pδPGKb	pBluescript II KS(+) derivative with homologous δ region, P_pgk1_, T_pgk1_, Bla^r^	Our lab
pδGAPz	pBluescript II KS(+) derivative with homologous δ region, P_gap_, T_pgk1_, Zeo^r^	Our lab
pδGAPh-CYS3	pδGAPh derivative with *S. cerevisiae CYS3*, HygB^r^	This study
pδGAPg-CYS4	pδPGKb derivative with *S. cerevisiae CYS4*, G418^r^	This study
pδPGKb-PDI	pδGAPz derivative with *S. cerevisiae PDI1*, Bla^r^	This study

### Effects of Acr on the growth behaviour of *S. cerevisiae* cells

Determinations of the initial concentration of Acr in the ALE experiments (Supplementary Table S1) and the effects of high-dosage Acr on the survival rate of the evolved strain (Supplementary Table S2) are described in detail in the supplementary File.

### Strain mutagenesis and adaptive evolutionary experiments

The prototrophic strain W303-1b/FGP^PT^ was pretreated using 50 μg/mL N-methyl-N′-nitro-N-nitrosoguanidine (MNNG), and the killing rate was ~90%. The MNNG-treated strain was designated W303-1b/FGP^PT^m. The ALE experiments were carried out as previously reported^11^, but with a slight modification (see the supplementary file).

### Extraction of metabolites and metabolomic analysis

The yeast metabolites were extracted using boiling ethanol (BE)^34^. The metabolic samples were analysed by relative quantitative measures (i.e., fold change across biological conditions) using an internal standard method. The mixture was detected using the “cell culture profiling” package on an LC-MS/MS system (Shimadzu, Japan). Fold changes were used to evaluate the specific metabolite in the evolved strain W303-1b/FGP^PT^-8–12 and the parental strain W303-1b/FGP^PT^ (Supplementary Table S3).

### Identification of Acr-thiol adducts and determination of Acr-GSH *in vivo*

The corresponding Acr-thiol adducts prepared as described in the supplementary file by *in vitro* chemical reactions were used as reference standards to monitor Acr-thiol adducts *in vivo* using LC-MS/MS.

## Electronic supplementary material

Supplementary Information
