# Supplementary material for: Acrolein-stressed threshold adaptation alters the molecular and metabolic bases of an engineered Saccharomyces cerevisiae to improve glutathione production
Source: Sci Rep. 2018 Mar 14;8:4506. doi: 10.1038/s41598-018-22836-2 (PMC5852114; doi:10.1038/s41598-018-22836-2)
Supplement: Supplementary file 1 — Supplementary Information [file 41598_2018_22836_MOESM1_ESM.pdf]

Supplementary files

Acrolein-stressed threshold adaptation alters the molecular and metabolic bases of an engineered *Saccharomyces cerevisiae* to improve glutathione production

Wenlong Zhou[1,+] ,Yan Yang[1,+], Liang Tang[2], Kai Cheng[3], Changkun Li[4], Huimin Wang[2], Minzhi Liu[2], Wei Wang[1,*]

[1] State Key Laboratory of Bioactive Substance and Function of Natural Medicines, Institute of Materia Medica, Peking Union Medical College & Chinese Academy of Medical Sciences, 1 Xian Nong Tan St., 100050 Beijing, China

[2] Key Laboratory of Biosynthesis of Natural Products of National Health and Family Planning Commission, Institute of Materia Medica, Peking Union Medical College & Chinese Academy of Medical Sciences, 1 Xian Nong Tan St., 100050 Beijing, China

[3] College of Life Science, Qufu Normal University, 273165 Qufu, Shandong, China

[4] Shimadzu (China) Co., Ltd. Beijing Branch, Chaoyangmen Wai St., 100020 Beijing, China

[*] Corresponding author: Wei Wang

Fax: 86-10-63017757

Phone number: 86-10-63165196

Email: wwang@imm.ac.cn

The First author: Wenlong Zhou, Yan Yang

Email: zhouwl@imm.ac.cn, yangyan@imm.ac.cn

List of tables

Table S1. Effects of Acr at different concentrations on the growth behavior of W303-1b/FGP^{PT} cells in WMVIII medium.

Table S2. Survival rates of the evolved strain W303-1b/FGP^{PT}-8-12 treated by different concentration of Acr

Table S3. Data used to generate the heat map of the metabolites.

Table S4. ^{1}H and ^{13}C NMR Chemical shift assignments of Acr-GSH.

Table S5. Primers used for cloning of *S. cerevisiae CYS3* gene.

Table S6. Primers used for cloning of *S. cerevisiae CYS4* gene.

Table S7. Primers used for cloning of *S. cerevisiae PDI1* gene.

Table S8. Primers used for cloning of *S. cerevisiae STR3* homologous DNA fragments.

Table S9. PCR primers used in the reverse mutation assay

List of figures

Fig. S1. Cell growth and GSH production of W303-1b/FGP^{PT} and W303-1b/FGP^{PT} in YPD medium.

Fig. S2. The stability of GSH production of W303-1b/FGP^{PT}, W303-1b/FGP^{PT}m, W303-1b/FGP^{PT}-8, W303-1b/FGP^{PT}-8-12 in 100 generations in WMVIII medium.

Fig. S3. GSH production analysis of single colonies of the evolved pools W303-1b/FGP^{PT} A-100 (A) and W303-1b/FGP^{PT}m A-75 (B).

Fig. S4. The selection of single colony with enhanced GSH production in YPD medium.

Fig. S5. ^{1}H NMR analysis of Acr-GSH in D_2O.

Fig. S6. GSH and Acr-GSH levels of W303-1b/FGP^{PT}-8-12 treated by 1.2 mM Acr.

Fig. S7. Metabolic changes involved in ATP generation, Cys and GSH biosynthesis.

Fig. S8. Chromatograms of samples from W303-1b/FGP^{PT} treated by sub-lethal and lethal dosages of Acr.

Fig. S9. Identification of Acr-thiol adducts *in vitro* by ESI mass spectrometry.

Fig. S10. Identification of Acr-thiol adducts *in vivo* by LC-MS/MS.

Fig. S11. Construction of integrative expression vectors pδGAPh-CYS3, pδGAPg-CYS4 and pδPGKb-PDI used to evaluate effects of overexpression of genes on the GSH production.

Fig. S12. Diagrammatic sketch of the knockout of cystathionine β-lyase genes via CRIPSR/Cas9-mediated gene editing method.

Fig. S13. Schematic diagram of the reversion of the auxotrophic strain.

Fig. S14. The sequencing results of reverse allelic sites.

Table S1. Effects of Acr at different concentrations on the growth behavior of W303-1b/FGP^{PT} cells in WMVIII medium[a].

Concentrations of Acr (mM)	0	0.1	0.2	0.4	0.6	0.8
OD_{600} at 48 h	74.1	56.9	27.9	0.2	0.2	0.2
OD_{600} at 96 h	83	71.5	62.8	44.7	0.2	0.2

[a] 0.2 mM Acr was selected as the initial concentration in the adaptive evolutionary experiments and as the sub-lethal dose to yeast cells. The lethal dose to the cells was 0.8 mM.

Table S2. Survival rates of the evolved strain W303-1b/FGP^{PT}-8-12 treated by different concentration of Acr.

Acr (mM)	Number of colonies			Cell survival (%)	*P* value
0	236	193	225	100	
0.8	194	213	216	95	0.26
1.0	204	219	197	95	0.24
1.2	205	191	207	92	0.16
1.4	188	194	212	91	0.13
1.6	183	198	201	89	0.09
1.8	189	204	182	88	0.08
2.0	195	181	197	88	0.08

Table S3. Data used to generate the heat map of the metabolites.

Metabolites	Fold changes														
	12 h			24 h			36 h			48 h			60 h		
Glucose	9.3[a]	11.7	10.1	1.3	1.2	1.2	1.4	1.2	1.3	1.6	1.8	2.1	1.2	1.1	1.1
Gluconic acid	2.0	1.6	1.7	0.7	1.2	1.1	2.3	1.5	1.8	1.7	1.8	2.5	2.4	2.4	2.5
Sucrose	0.4	0.6	0.5	1.5	1.6	1.5	1.5	1.3	1.6	1.4	1.5	1.5	1.6	1.5	1.6
Cystine	1.7	1.4	1.5	4.0	2.3	3.8	1.4	1.1	1.9	1.5	1.6	1.6	2.7	2.7	3.5
Asparagine	1.6	1.6	1.6	0.8	1.3	1.2	1.3	1.2	1.5	1.0	1.0	1.0	1.2	1.3	1.2
Aspartic acid	2.2	2.4	2.2	0.5	1.3	1.2	1.4	1.3	1.5	0.3	0.3	0.2	0.4	0.5	0.5
Serine	1.9	1.8	1.7	0.6	2.3	1.7	2.1	1.7	2.5	2.7	2.9	3.2	5.0	5.7	5.4
Cystathionine	5.7	6.0	5.6	1.0	5.1	4.4	5.1	4.2	6.3	11.5	10.6	8.8	12.8	12.7	14.0
Glycine	1.3	1.0	1.1	0.5	2.3	1.7	8.2	5.5	8.0	5.3	6.2	5.0	14.6	11.6	11.6
Glutamine	2.0	2.0	1.9	1.5	0.7	0.4	1.9	1.4	2.0	1.4	1.4	1.7	1.0	0.9	0.9

Cysteine	2.0	2.5	2.2	2.5	3.0	3.1	16.4	9.3	14.9	9.7	9.2	14.8	1.7	1.9	2.2
Threonine	2.0	2.0	1.9	2.5	0.9	0.9	3.5	2.2	3.5	1.8	1.8	2.3	2.4	2.1	2.1
Methionine sulfoxide	2.6	2.4	2.4	1.2	0.9	0.8	2.8	1.3	1.8	0.4	0.6	0.7	2.7	2.0	2.0
Glutamic acid	1.3	1.4	1.3	1.6	0.7	0.7	1.4	1.3	1.6	1.2	1.2	1.1	1.7	1.6	1.6
Alanine	1.7	1.6	1.6	1.3	0.2	0.1	2.1	1.7	2.2	0.9	1.1	1.1	3.4	3.0	3.0
Ornithine	1.2	1.2	1.2	1.5	2.6	2.3	8.1	7.6	8.9	16.5	17.3	17.2	14.0	14.5	14.7
Proline	2.0	2.2	2.0	0.8	1.7	1.6	4.1	3.5	4.8	1.2	1.3	1.5	1.6	1.6	1.6
Histidine	1.5	1.6	1.5	3.1	2.1	2.1	2.4	2.0	2.8	2.3	2.4	2.4	2.7	2.7	2.7
2-Aminoadipic acid	16.6	14.0	14.8	0.8	0.7	0.6	0.7	0.7	0.8	4.0	6.7	3.3	3.0	2.9	3.3
Arginine	0.4	0.5	0.4	0.5	0.8	0.7	1.0	0.9	1.1	1.3	1.3	1.3	1.4	1.4	1.4
N-Acetylaspartic acid	1.9	1.8	1.8	0.7	1.0	0.9	1.3	1.2	0.8	1.7	1.7	1.8	1.4	1.3	2.0
4-Aminobutyric acid	0.1	0.2	0.1	0.3	0.7	0.6	0.6	0.5	0.7	1.7	1.8	2.2	1.8	1.6	2.0
Methionine	1.4	1.3	1.3	0.6	0.7	0.7	1.2	0.9	1.1	0.9	1.4	1.9	2.4	1.9	1.5

N-Acetylcysteine	4.7	5.4	4.9	5.2	7.0	6.7	6.0	5.0	5.2	10.3	10.0	16.6	2.7	3.9	4.1
Oxidized glutathione	1.1	1.3	1.2	1.2	1.4	1.3	1.4	1.3	1.5	1.5	1.6	1.6	1.6	1.7	1.6
Tyrosine	1.2	1.2	1.2	2.4	2.1	2.0	1.4	1.3	1.5	0.9	1.0	1.3	1.5	1.5	1.5
Phenylalanine	1.4	1.4	1.3	1.4	1.5	1.4	1.3	1.2	1.3	1.1	1.2	1.3	1.4	1.5	1.5
Kynurenine	4.6	4.9	4.6	1.4	1.7	1.6	1.4	1.0	1.2	3.5	3.5	7.8	2.6	3.1	2.9
Tryptophan	1.3	1.3	1.3	1.8	1.7	1.6	1.4	1.2	1.4	1.0	1.2	1.5	1.9	2.0	2.0
Homocysteine	2.0	2.1	1.9	2.5	0.2	0.2	1.7	1.6	1.9	4.4	2.9	4.3	2.3	2.1	2.0
Homoserine	1.9	1.8	1.8	2.5	1.2	0.8	3.3	2.5	3.5	1.8	1.8	2.3	2.6	2.2	2.2
Cytidine monophosphate	0.7	0.7	0.7	1.7	3.8	3.4	1.7	1.8	2.0	1.1	1.4	1.5	1.1	/	1.2
Adenosine monophosphate	1.2	1.3	1.2	1.2	1.8	1.7	1.2	1.6	1.8	1.2	1.5	1.6	1.5	1.4	1.4
Uridine	1.7	1.7	1.7	2.3	2.0	1.9	2.0	1.7	2.1	1.9	2.4	2.7	7.7	7.8	7.3

Thymine	4.6	4.9	4.5	0.6	1.5	1.3	0.8	0.8	0.8	2.5	3.4	3.8	5.7	5.8	6.5
Inosine	1.4	1.4	1.4	1.6	2.0	1.7	1.8	1.0	1.0	0.8	1.9	2.6	1.1	1.6	1.1
Guanosine	0.8	0.9	0.8	2.7	1.9	1.7	2.7	2.1	2.6	0.9	1.1	1.6	1.6	1.8	1.5
Xanthosine	1.0	1.0	1.0	2.9	2.0	1.8	3.2	2.0	2.4	0.7	0.9	1.4	1.6	2.1	1.9
Cytidine	1.2	1.2	1.2	0.6	0.9	0.8	1.6	0.2	0.9	0.2	0.3	0.4	0.5	0.4	0.5
Thymidine	1.7	1.3	1.4	1.8	1.4	1.3	2.1	1.5	1.5	1.0	1.3	1.2	1.7	2.1	2.0
Adenine	3.2	3.1	3.0	0.7	1.0	1.0	3.5	2.2	2.5	0.8	1.5	2.3	1.2	1.4	1.2
Adenosine	1.4	1.4	1.3	1.3	1.5	1.3	1.8	1.4	1.6	1.0	1.3	1.5	1.5	1.6	1.5
Deoxycytidine	0.9	0.7	0.8	1.2	1.6	1.5	2.0	1.2	1.5	0.7	0.8	0.9	0.8	0.7	0.7
Nicotinic acid	0.7	1.2	0.9	/[b]	0.6	0.5	/	/	/	/	/	3.1	1.7	1.8	1.8
Pyridoxal	1.6	1.5	1.5	0.6	0.6	0.6	1.7	1.4	1.7	5.2	5.4	6.8	2.2	2.7	2.7
4-Aminobenzoic acid	1.1	0.8	0.9	1.8	1.5	1.6	1.0	1.0	1.2	2.6	2.1	2.4	1.3	1.3	1.3
Riboflavin	1.5	1.5	1.5	2.6	2.4	2.3	2.0	1.8	1.9	1.5	1.3	1.6	1.9	2.6	2.2

Biotin	1.4	1.5	1.4	0.6	0.7	0.6	0.4	0.3	0.3	0.2	0.3	0.4	0.8	0.8	0.7
Tocopherol acetate	1.5	1.5	1.4	1.1	1.1	1.2	1.3	1.1	2.6	1.2	1.4	1.3	1.5	1.5	1.5
O-Phosphoethanolamine	1.5	1.4	1.4	0.7	0.5	0.5	0.9	0.9	1.1	2.5	2.7	2.8	1.8	1.9	13.0
Glyceric acid	1.8	1.3	1.5	1.5	1.0	1.0	0.9	0.9	1.0	1.1	1.0	1.5	1.4	1.2	1.2
Malic acid	0.6	0.7	0.6	0.2	0.6	0.5	0.5	0.4	0.6	2.3	2.2	1.8	2.5	2.2	2.2
Isocitric acid	2.0	2.1	2.0	/	/	/	/	1.3	/	2.0	2.1	1.8	1.4	1.4	1.5
Pyruvic acid	1.7	2.1	1.8	/	/	/	2.0	1.5	1.8	0.2	0.5	0.6	3.8	5.2	5.2
2-Aminoethanol	1.9	1.5	1.6	1.5	1.7	1.7	4.0	3.6	3.9	4.9	6.1	7.7	4.3	4.8	4.8
Lactic acid	1.2	1.1	1.1	0.3	0.4	0.4	1.1	0.7	0.9	0.1	0.3	0.3	0.7	0.7	0.7
Citric acid	1.9	1.9	1.8	1.7	3.1	2.9	2.0	1.8	2.2	1.2	1.3	1.2	1.4	1.4	1.5
Putrescine	0.6	0.6	0.6	0.5	1.2	1.0	0.5	0.5	0.6	1.8	1.7	1.3	1.5	1.9	1.8
Fumaric acid	0.6	0.7	0.6	0.3	0.3	0.3	0.5	0.4	0.5	1.0	1.1	1.0	1.4	1.4	1.4
2-Ketoisovaleric acid	1.0	1.1	1.0	1.0	0.8	0.9	0.8	0.6	0.9	0.4	0.5	0.4	1.0	0.6	1.0

4-Hydroxyphenyllactic acid	1.5	1.7	1.5	4.3	2.2	2.1	3.0	2.8	2.6	1.1	1.1	1.1	2.6	2.6	2.6
3-Methyl-2-oxovaleric acid	1.3	2.3	1.7	1.9	1.3	1.2	5.0	4.0	4.3	1.2	1.2	1.3	0.8	0.6	0.7

[a] Metabolites of triplicate samples isolated from each set which was removed at the given intervals were detected, respectively; [b] No MS intensity detected, 1.0-fold change was used to generate the heat map.

Table S4. ^{1}H and ^{13}C NMR Chemical shift assignments of Acr-GSH.

proton no.		carbon no.	
1 (*H*C=O)	9.66 s	1 (HC=O)	206
1' [C*H*(OH)$_2$]	5.13 t	1' [CH(OH)$_2$]	89.7
2 (CH$_2$)	1.88 q	2 (CH$_2$)	42.8
3 (CH$_2$)	2.65 t	2 ' (CH$_2$)	36.8
5 (CH$_2$)	3.08 dd	3 (CH$_2$)	26.9
	2.89 dd	5 (CH$_2$)	32.9
6 (CH)	4.58 br dd	6 (CH)	53.1
7 (NH)		8 (C=O)	173.8
9 (CH$_3$)	2.17 dd	9 (CH$_3$)	26.1
10 (CH$_2$)	2.54 m	10 (C=O)	31.3
11 (CH)	3.81 t	11 (CH)	53.9
15 (CH$_2$)	3.96 s	12 (C=O)	174.9
		13 (C=O)	173.7
		15 (CH$_2$)	41.7
		16 (C=O)	172.8

Table S5. Primers used for cloning of *S. cerevisiae CYS3* gene (GenBank No. NM_001178157).

Primers	Oligonucleotide sequences (5'-3')
CYS3_1	GAGGCCTATACACATAGACATTTGC
CYS3_2	ACACATAAACAAACAAACATATGACTCTACAAGAATCTGATAAAT
CYS3_3	CAATTCAATTCAATGCTAGCTTAGTTGGTGGCTTGTTTCAAGGCTTG
CYS3_4	AAGGTCCGGTCGAAGGCAGAGACGTGG

Table S6. Primers used for cloning of *S. cerevisiae CYS4* gene (GenBank No. NM_001181284).

Primers	Oligonucleotide sequences (5'-3')
CYS4_1	CGTTGTAGGCCACTTGCTCAAAGGA
CYS4_2	ACACATAAACAAACAAACATATGACTAAATCTGAGCAGCAAGCCG
CYS4_3	CAATTCAATTCAATGCTAGCTTATGCTAAGTAGCTCAGTAAATCCAT
CYS4_4	AATGACGGATTTTGCTTCTATGTTTGC

Table S7. Primers used for cloning of *S. cerevisiae PDI1* gene (GenBank No. NM_001178688).

Primers	Oligonucleotide sequences (5'-3')
PDI_P1	GCCAAGCTCTACATAAAGAAAAACATA
PDI_P2	TTACAACAAATATAAAACATATGAAGTTTTCTGCTGGTGCCGTCCTG
PDI_P3	TTCAATTCAATTCAATGCTAGCTTACAATTCATCGTGAATGGCATCTTC
PDI_P4	CAACTATTGTGTTTGAATTTTAACGTTTA

Table S8. Primers used for cloning of *S. cerevisiae STR3* (GenBank NO. NM_001181049) homologous DNA fragments.

Primers	Oligonucleotide sequences (5'-3')
STR3_1	CGTTGTAGGCCACTTGCTCAAAGGA
STR3_2	ACACATAAACAAACAAACATATGACTAAATCTGAGCAGCAAGCCG
STR3_3	CAATTCAATTCAATGCTAGCTTATGCTAAGTAGCTCAGTAAATCCAT
STR3_4	AATGACGGATTTTGCTTCTATGTTTGC
STR3_G1	[b] ***CTCATCATAGTATTATCAAC***GATCATTTATCTTTCACT
STR3_G2	***GTTGATAATACTATGATGAG***GTTTTAGAGCTAGAAATA
STR3_H1[a]	AAGTTGATTGTGTTCTTCTAGAGTCTCCGACCAATCCGCTTTGCATTT ATCATTATCAATACTGCCATTT
STR3_H2[a]	GATAATCCAGCTCCTGTAGAATTAATGACAAAGTAAAGCTTCGAGAC GCAAGGATTGATAATGTAATAGG

[a] Primers STR3_H1 and STR3_H2 were used for the amplification of the homologous

integration fragment of STR3 to disrupt it. Homologous fragments of STR3 gene were underlined. [b] sequence of genes encoding gRNA were black bold.

Table S9. PCR primers used in the reverse mutation assay.

Primer name	Primer sequence (5' - 3')	Size (bp)
PGK1_Sph	TTCGAAGCATGCGAAGTACCTTCAAAGAATG	31
PGK1_Nde	CGACCGTACGTCTGGATCCGTTTAAACGCATATGTTTGT TTTATATTTGTTGTAAAA	57
X260_ 1	AACTCCCATATGGACAAGAAGTACAGCATCGGCCTG	36
X260_2	CTATATCGAATTCGAGCTCGTCCACCACCTTC	32
X260_3	TGGTGGACGAGCTCGTGAAA	20
X260_4	GAATTCCTCGAGTCACACCTTCCTCTTCTTCTTGGGGTCG TCGCCTCCCAGCTGAGAC	58
SNR_G1	TTGCTTGCATGCTCTTTGAAAAGATAATGTATGATTATGCT TTCACTCATATTTATACA	59
SNR _G2	TTTCACTCATATTTATACAGAAACTTGATGTTTTCTTTCGA GTATATACAAGGTGATTA	59
SNR _G3	GTATATACAAGGTGATTACATGTACGTTTGAAGTACAACT CTAGATTTTGTAGTGCCCT	59
SNR _G4	GTAGGGTGTGAAAAAATGCGCACCTTTACCGCTAGCCCA AGAGGGCACTACAAAATCTA	59
SNR _G5	ACTTCTACAGCGTTTGACCAAAATCTTTTGAACAGAACA	59

	TTGTAGGGTGTGAAAAAATG	
SNR _G6	GCGGAGAAGTTTCGAACGCCGAAACATGCGCACCAACT TTCACTTCTACAGCGTTTGAC	59
SNR _G7	GATCATTTATCTTTCACTGCGGAGAAGTTTCGAACGCCG	39
ADE_G1	[a]TTAACGTGGTCATTGGAGTTGATCATTTATCTTTCACT	38
ADE_G2	AACTCCAATGACCACGTTAA*G*TTTTAGAGCTAGAAATAG CAAGTT	45
SNR _G8	GAGCTAGAAATAGCAAGTTAAAATAAGGCTAGTCCGTTAT CAACTTGAAAAAGTGGCAC	59
SNR _G9	AATCCTGTTAACAAAAAAACAAAAAAAGCACCACCGAC TCGGTGCCACTTTTTCAAGTT	59
GADT7_H3	TTACGAGGGCTTATTCAGAAGCTT	24
GADT7_Nhe	TCGACCTGCAGCCAAGCTAGCGGCCGGTAGAGGTGTGG TCA	41
Bla_Nhe	TGACCACACCTCTACCGGCCGCTAGCCCCACACACCATA GCTTCAA	46
Bla_Not	TTATATTAAGGGTTCCGGATCGCGGCCGCAGCTTGCAAA TTAAAGCCTT	49
Ura_G1	GTAGAGACCACATCATCCACGATCATTTATCTTTCACT	38
Ura_G2	GTGGATGATGTGGTCTCTAC*G*TTTTAGAGCTAGAAATA	38
Leu_G1	TCACCATCGTCTTCCTTTCTGATCATTTATCTTTCACT	38
Leu_G2	AGAAAGGAAGACGATGGTGA*G*TTTTAGAGCTAGAAATA	38

Trp_G1	ACCAAGTATTTCGGAGTGCCGATCATTTATCTTTCACT	38
Trp_G2	GGCACTCCGAAATACTTGGT*G*TTTTAGAGCTAGAAATA	38
His_G1	ATGCACTCAACGATTAGCGAGATCATTTATCTTTCACT	38
His_G2	TCGCTAATCGTTGAGTGCAT*G*TTTTAGAGCTAGAAATA	38
Ade1[b]	ATGGATTCTAGAACAGTTGGTATA	24
Ade2[b]	CATTCAATAGGGACGTCTCACTGGC	25
Ura1[b]	ATGTCGAAAGCTACATATAAGGAAC	25
Ura2[b]	TTTGTGAGTTTAGTATACATGCATTTAC	28
Leu1[b]	ATGTCTGCCCCTAAGAAGATCGTC	24
Leu2[b]	TTAAGCAAGGATTTTCTTAACTTCT	25
Trp1[b]	ATGTCTGTTATTAATTTCACAGGT	24
Trp2[b]	CTATTTCTTAGCATTTTTGACGAAA	25
His1[b]	ATGACAGAGCAGAAAGCCCTAGTAA	25
His2[b]	CTACATAAGAACACCTTTGGTGGAG	25
Ade3[c]	GACAATTGGGACGTATGATTGT	22
Ura3[c]	GATGTTAGCAGAATTGTCATGC	22
Leu3[c]	CAGGTGACCACGTTGGTCAAGA	22
Leu4[c]	CTTAACTTCTTCGGCGACAGCA	22
Trp3[c]	GTAGTTCTGGTCCATTGGTGAA	22
His3[c]	GCGTATTACAAATGAAACCAAG	22

[a] sequence of genes encoding gRNA were underlined; [b] primers used for PCR amplification the alleles; [c] primers used for the DNA sequencing.

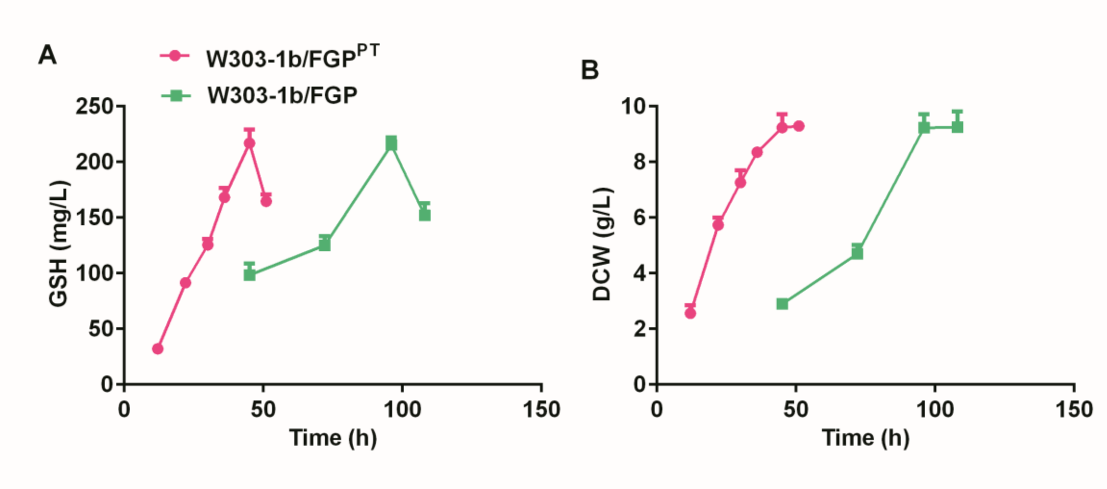

Fig. S1. Cell growth and GSH production of W303-1b/FGP and W303-1b/FGP^{PT} in the YPD medium. (A) DCW; (B) GSH concentration. The values are presented as the means, and the error bars show the SD (n=3).

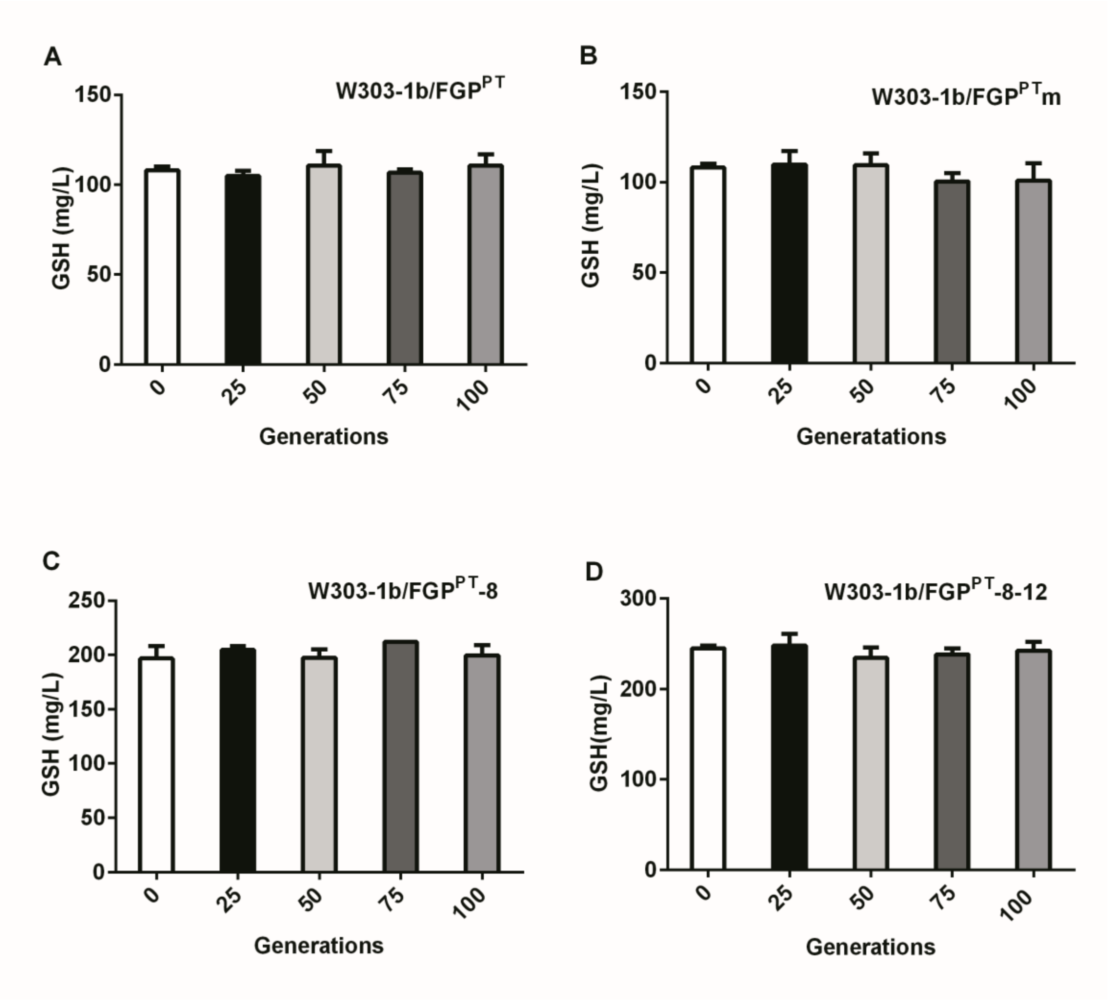

Fig. S2. The stability of GSH production of W303-1b/FGP^{PT}, W303-1b/FGP^{PT}m, W303-1b/FGP^{PT}-8, W303-1b/FGP^{PT}-8-12 in 100 generation in WMVIII medium. (A) W303-1b/FGP^{PT}; (B) W303-1b/FGP^{PT}m; (C) W303-1b/FGP^{PT}-8; (D) W303-1b/FGP^{PT}-8-12. All strains were inoculated in WMVIII medium with an initial OD_{600} of 0.5. The cells were transferred into fresh medium when grown to mid-exponential growth phase (OD_{600} = 15-30). An aliquot of the yeast cultures was stored at each transfer for detection of GSH production. The values are presented as the means, and the error bars show the SD (n=3).

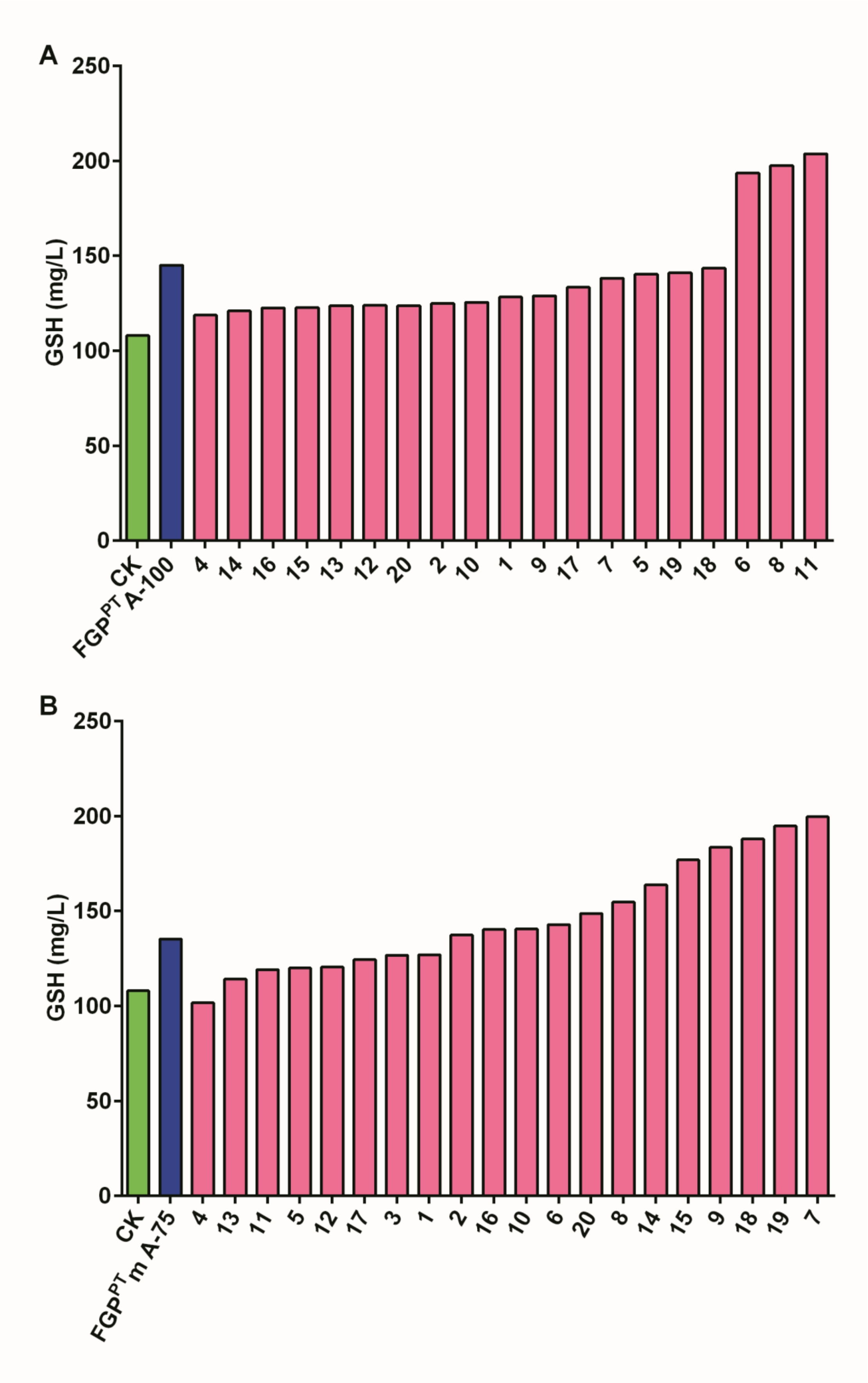

Fig. S3. GSH production analysis of single colonies of the evolved pools

W303-1b/FGP^{PT} A-100 (A) and W303-1b/FGP^{PT}m A-75 (B). The isolates were numbered from 1 to 20 with arrangement in the figure according to their GSH production. CK in (A) and (B) represent the GSH production of W303-1b/FGP^{PT} A and W303-1b/FGP^{PT}m, respectively. The columns of FGP^{PT} A-100 and FGP^{PT} m A-75 represent GSH production of populations W303-1b/FGP^{PT} A-100 and W303-1b/FGP^{PT} m A-75 obtained after the first round of adaptive evolution experiment, respectively.

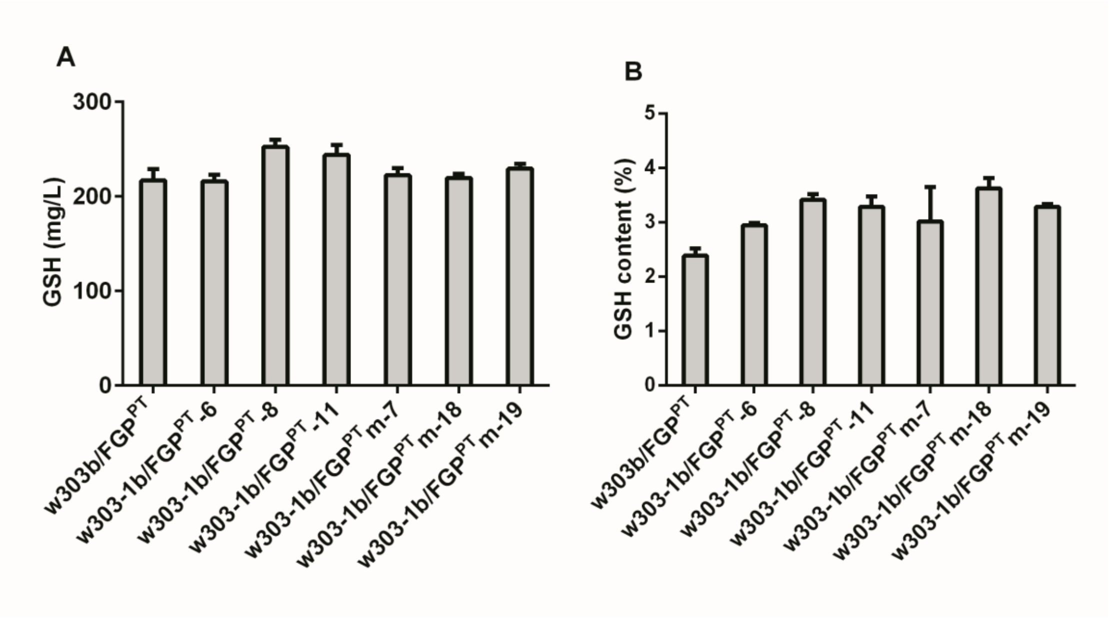

Fig. S4. The selection of single colony with enhanced GSH production in YPD medium. (A) GSH production (mg/L), (B) GSH content (%). Six colonies of the evolved populations W303-1b/FGP^{PT}m A-75 and W303-1b/FGP^{PT} A-100 were selected in a 250-ml conical flask containing 50-ml YPD medium for 48 h. An aliquot of yeast cultures was stored at each transfer for the detection of GSH production. The values are presented as the means, and the error bars show the SD (n=3).

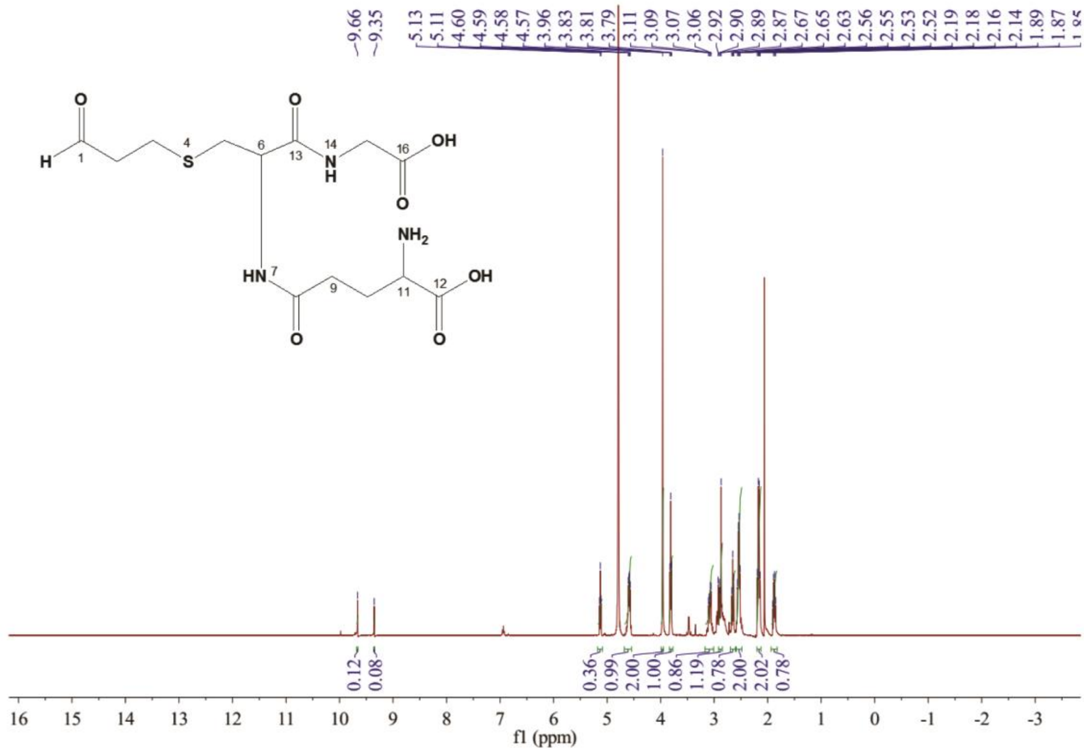

Fig. S5. ^{1}H NMR analysis of Acr-GSH in D_2O.

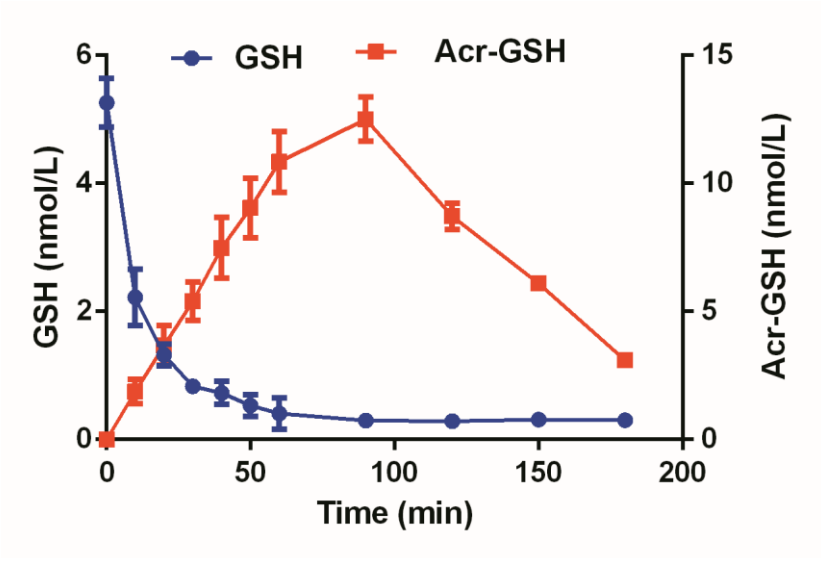

Fig. S6. GSH and Acr-GSH levels of W303-1b/FGP[PT]-8-12 treated by 1.2 mM Acr. Levels of GSH were measured by HPLC, while Acr-GSH were measured by LC-MS/MS. Data represent means and S.E.M. from three independent experiments

performed with multiple estimations per point. When not shown, error bars lie within the symbols.

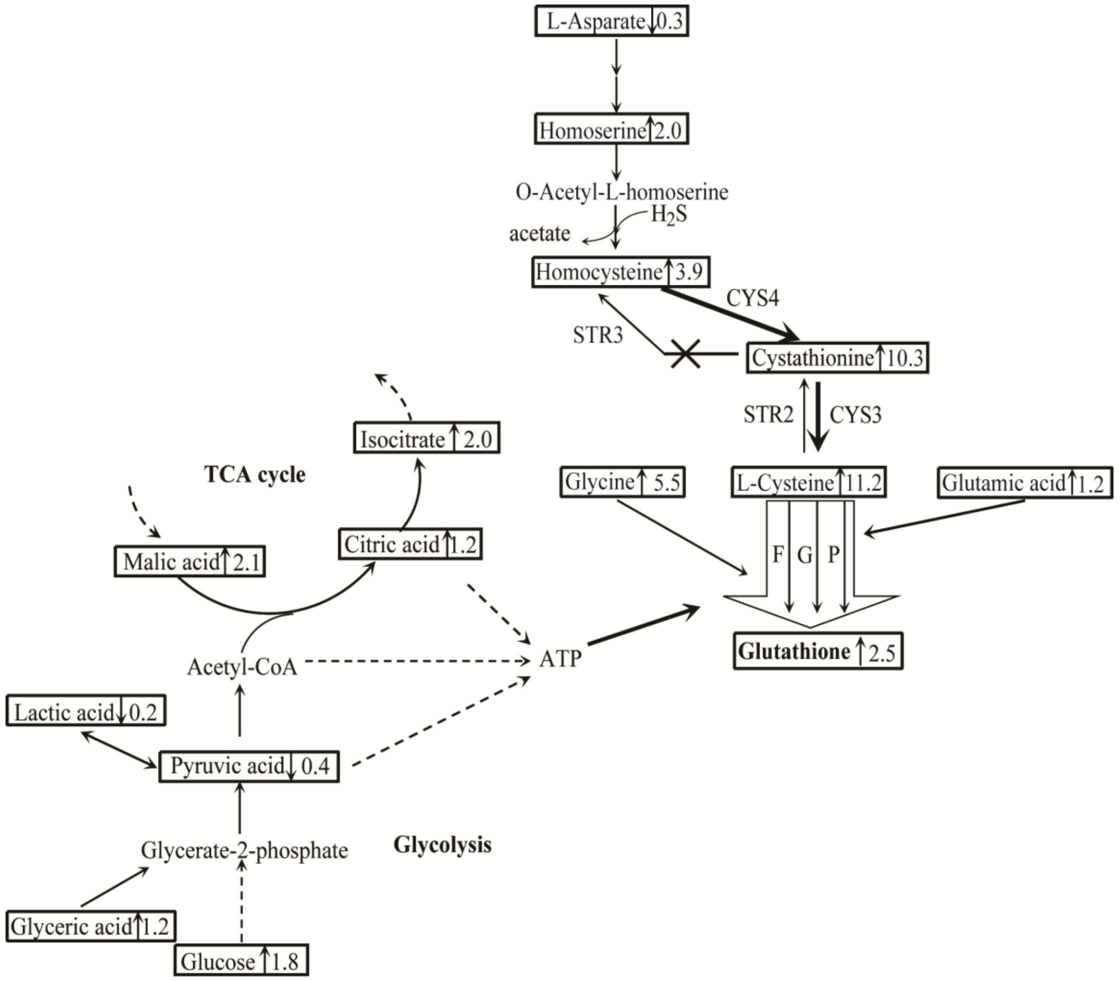

Fig. S7. Metabolic changes involved in ATP generation, Cys and GSH biosynthesis. Precursor supplement and ATP regeneration promoted the enhancement of GSH production of the evolved strain W303-1b/FGP^{PT}-8-12. The levels of GSH precursors, especially that of Cys, and intermediates involved in Cys biosynthetic pathway were higher in the evolved strain than that in W303-1b/FGP^{PT}. The levels of metabolites involved in energy metabolism were also changed.

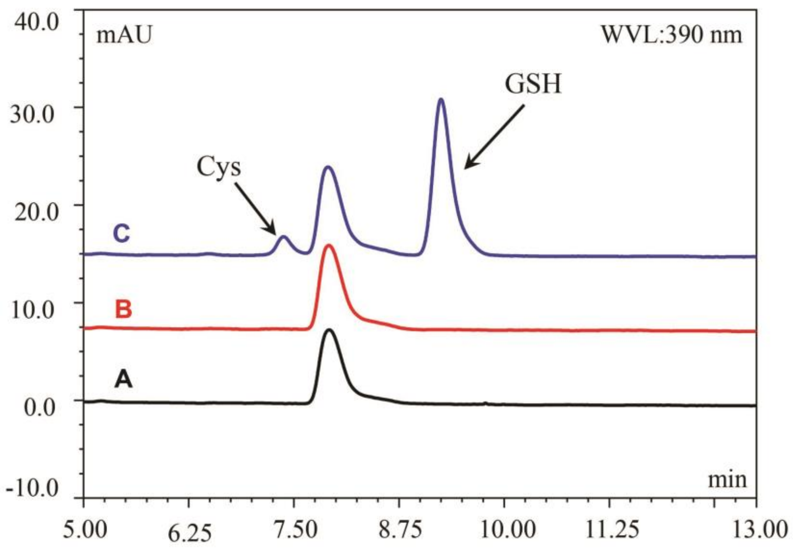

Fig. S8. Chromatograms of samples from W303-1b/FGP^{PT} treated by sub-lethal and lethal dosages of Acr. A. lethal dosages; B. sub-lethal dosages; C. samples from W303-1b/FGP^{PT} without treatment. W303-1b/FGP^{PT} was cultured in WMVIII medium with an initial OD_{600} of 0.2 for 36 h. Different concentrations of Acr were added and the cells were cultured for additional 1 h in phosphate-buffered saline (PBS). After extraction and derivatization, the GSH and Cys of the samples were measured by HPLC analysis.

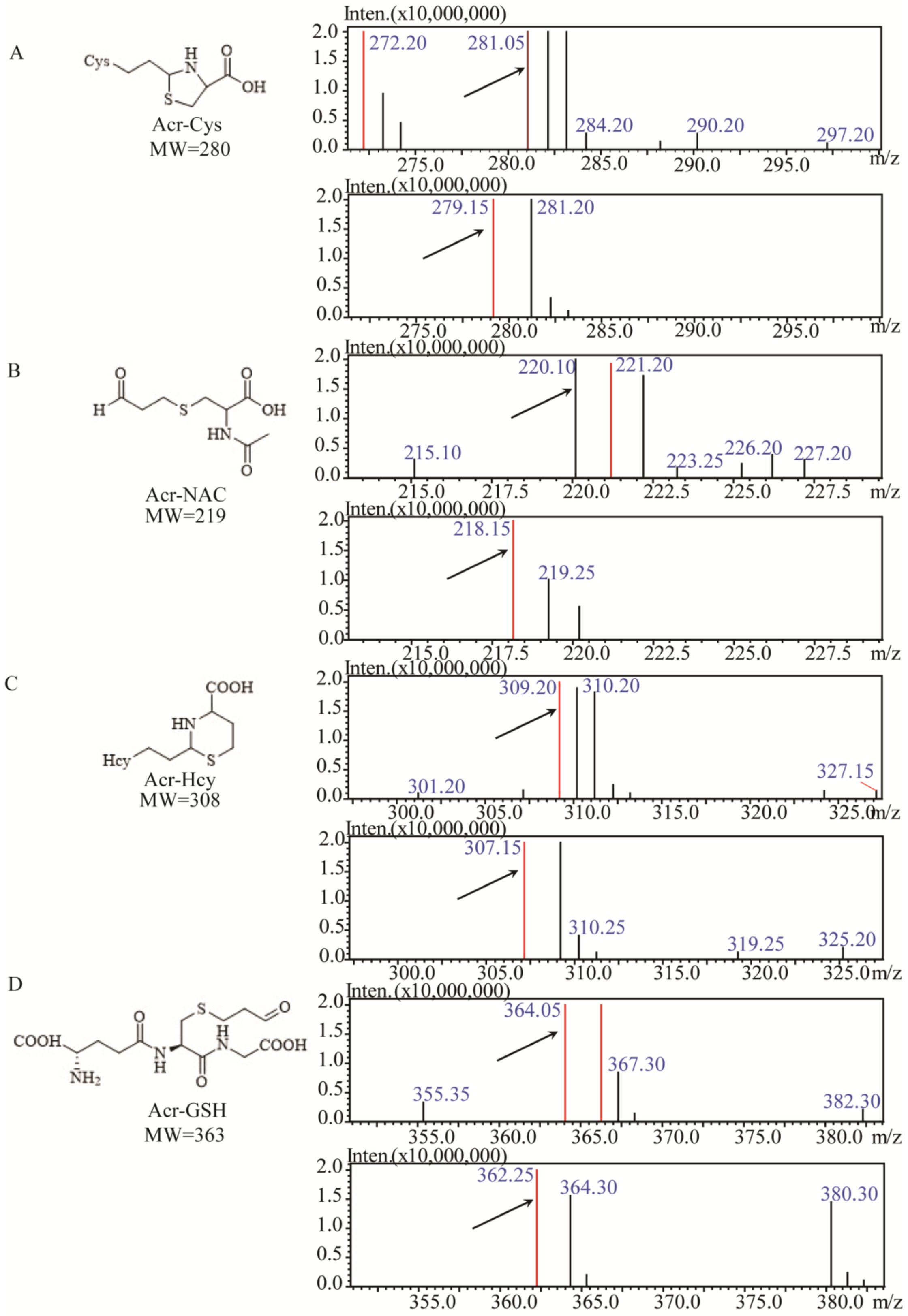

Fig. S9. Identification of Acr-thiol adducts *in vitro* by ESI mass spectrometry. A. Acr-Cys; B. Acr-NAC; C. Acr-Hcy; D. Acr-GSH.

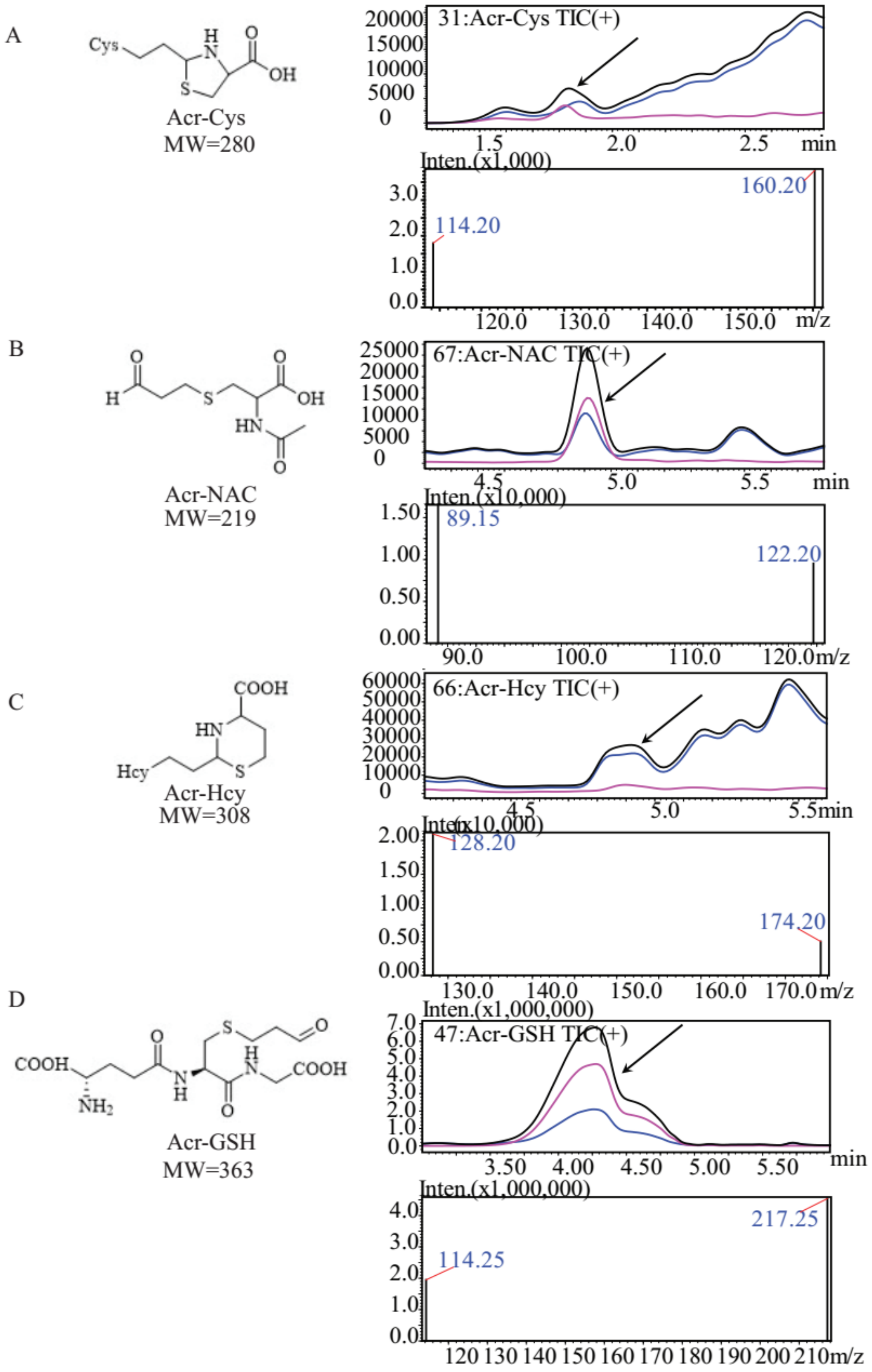

Fig. S10. Identification of Acr-thiol adducts *in vivo* by LC-MS/MS. A. Acr-Cys; B.

Acr-NAC; C. Acr-Hcy; D. Acr-GSH. W303-1b/FGP^{PT} was cultured in WMVIII medium with an initial OD_{600} of 0.2 for 36 h. Sub-lethal dosages of Acr were added and the cells were cultured for additional 1 h in PBS. After quenching and extraction of the samples, Acr-thiol adducts were identified.

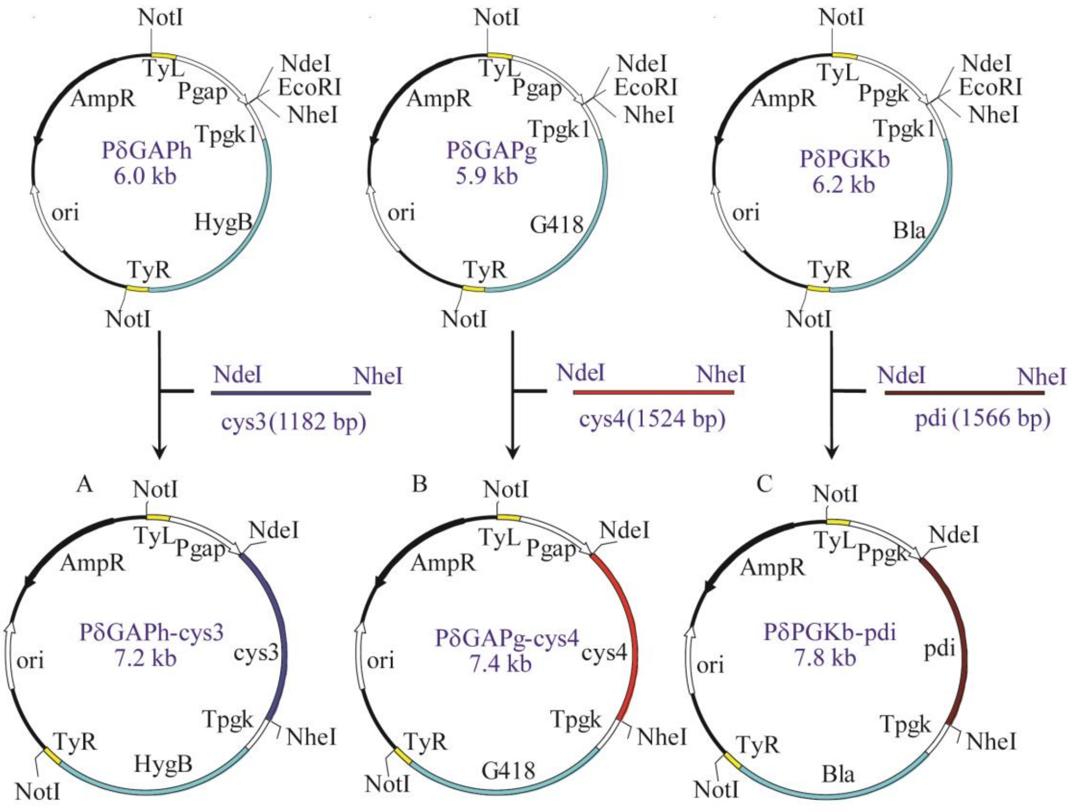

Fig. S11. Construction of integrative expression vectors pδGAPh-CYS3, pδGAPg-CYS4 and pδPGKb-PDI used to evaluate effects of overexpression of genes on the GSH production. The DNA fragment of *CYS3* gene was obtained by polymerase chain reaction using genome DNA as a template and inserted into plasmid pδGAPh constructed previously in our lab, generating the expression vector pδGAPh-CYS3 (A). Following the construction of plasmid pδGAPh-CYS3, the expression vectors pδGAPg-CYS4 (B) and pδPGKb-PDI (C) were constructed.

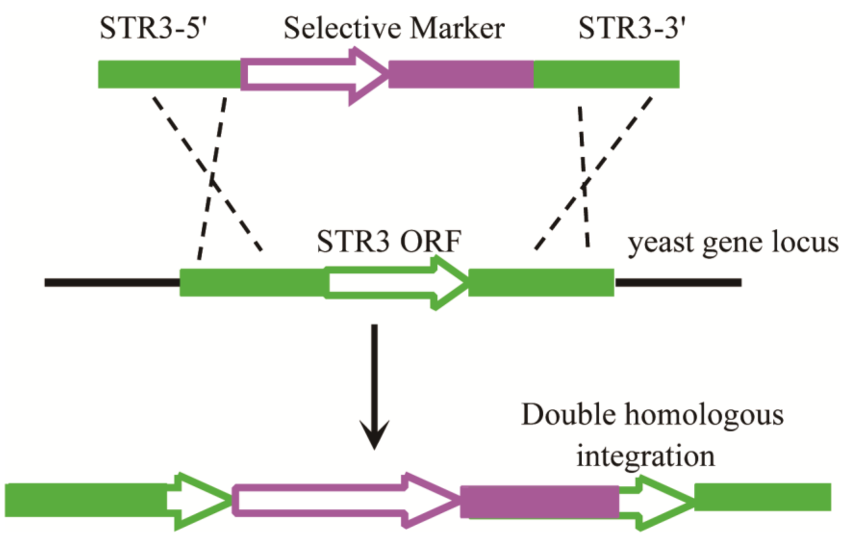

Fig. S12. Diagrammatic sketch of the knockout of cystathionine β-lyase genes via CRIPSR/Cas9-mediated gene editing method. The STR3-5' and STR3-3' represent the homologous DNA fragments targeted *STR3* gene. Antibiotic *zeocin* resistance (*Sh ble*) gene was used to select the deletion of *STR3*. The synthesis of gRNA expression cassette and the disruption of *STR3* gene was performed as described in the autotrophic reversion assay.

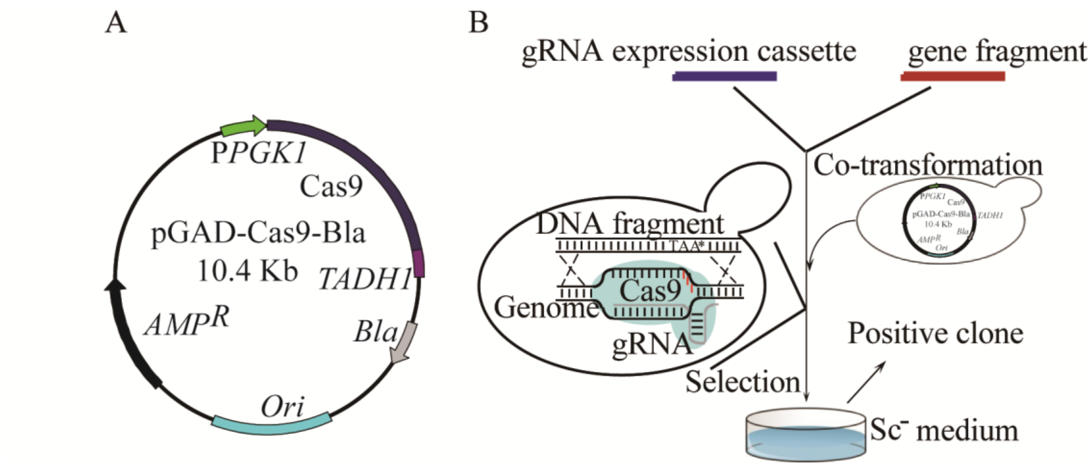

Fig. S13. Schematic diagram of the reversion of the auxotrophic strain (A: Schematic diagram of pGAD-Cas9-Bla; B: The reversion of the auxotrophic strain mediated by CRISPR/Cas9).

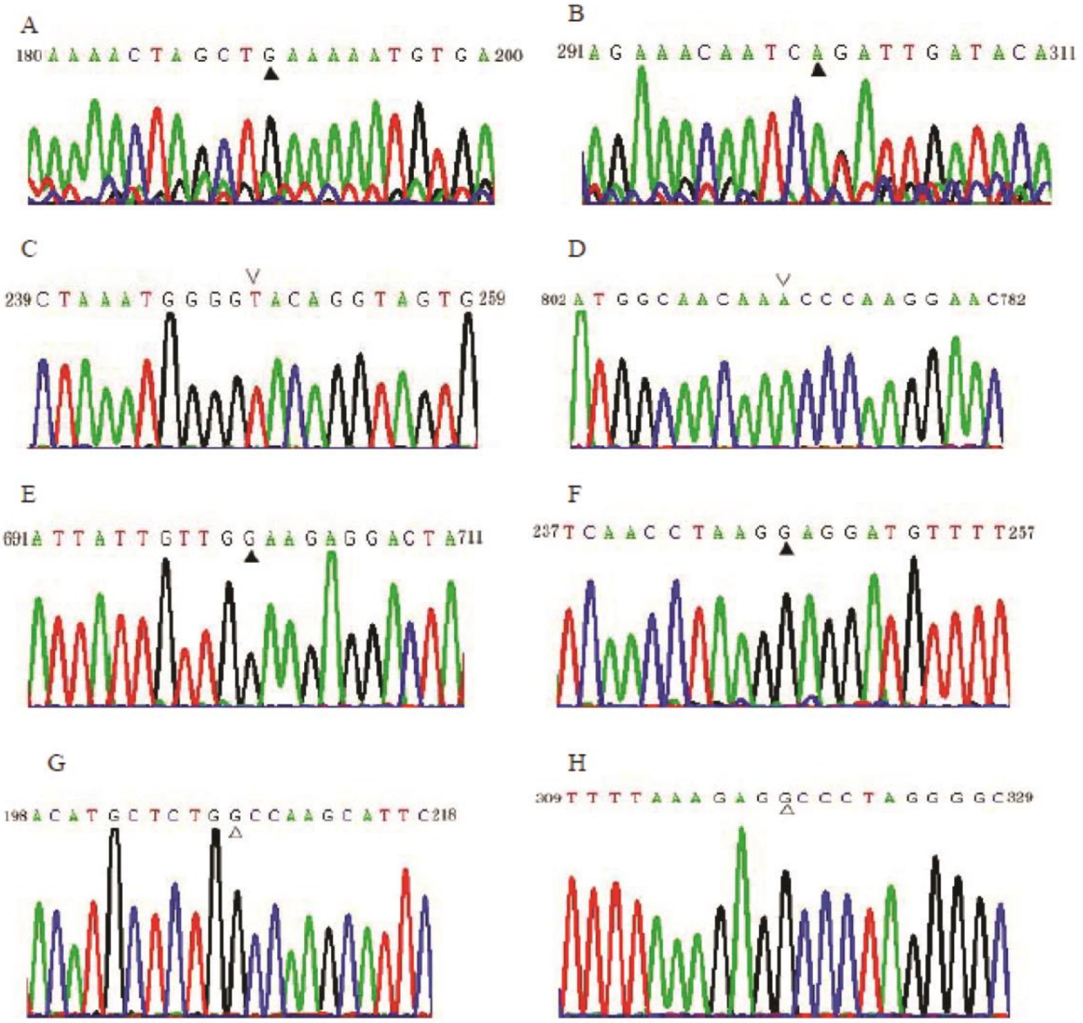

Fig. S14. The sequencing results of reverse allelic sites. ▲: reverse mutation. ∨: reverse insertion; △: reverse deletion. (A) ade2-1 T190G, reverse ochre mutation; (B) ade2-1 G301A, Gly>Arg reverse mutation; (C) Leu2-3,112, G249 reverse an insertion; (D) Leu2-3,112, G792 reverse an insertion; (E) ura3-1 A701G, Glu>Gly reverse mutation; (F) trp1-1 T190G, reverse amber mutation; (G) his3-11,15 G208 reverse deletion; (H) his3-11,15 G319 reverse deletion.

Supplementary Methods

Plasmid construction and yeast transformation

Cloning of *S. cerevisiae CYS3* and construction of expression vector pδGAPh-CYS3. Based on the encoding sequence of *S. cerevisiae CYS3* (GenBank No. NM_001178157), the synthesized primers (Supplementary Table S5) were used to clone the whole DNA fragment of *CYS3*.

DNA fragment of *CYS3* was amplified by Nested-PCR. Primers CYS3_1 and CYS3_4 were used for the first-round amplification with the genomic DNA of *S. cerevisiae* W303-1b as a template. Primers CYS3_2 and CYS3_3 were used for the second-round amplification with the product of the first-round PCR as the template. The obtained CYS3 fragment was inserted into plasmid pδGAPh digested by *Nde* I and *Nhe* I, generating plasmid pδGAPh-CYS3.

Cloning of S. *cere*visiae *CYS4* and construction of expression vector pδGAPg-CYS4. Based on the encoding sequence of *S. cerevisiae CYS4* (GenBank No. NM_001181284), the synthesized primers (Supplementary Table S6) were used to clone the whole DNA fragment of *CYS4*.

DNA fragment of *CYS4* was amplified by Nested-PCR. Primers CYS4_1 and CYS4_4 were used for the first-round amplification with the genomic DNA of *S. cerevisiae* W303-1b as a template. Primers CYS4_2 and CYS4_3 were used for the second-round amplification with the product of the first-round PCR as the template. The obtained CYS4 fragment was inserted into plasmid pδGAPg digested by *Nde* I and *Nhe* I, generating plasmid pδGAPg-CYS4.

Cloning of *S. cere*visiae *PDI1* and construction of expression vector pδGAPb-PDI. Based on the encoding sequence of *S. cerevisiae PDI1* (GenBank No. NM_001178688), the synthesized primers (Supplementary Table S7) were used to clone the whole DNA fragment of *PDI1*.

DNA fragment of *PDI1* was amplified by Nested-PCR. Primers PDI_P1 and PDI_P4 were used for the first-round amplification with the genomic DNA of *S. cerevisiae* W303-1b as a template. Primers PDI_P2 and PDI_P3 were used for the second-round amplification with the product of the first-round PCR. The obtained *PDI1* DNA fragment was inserted into plasmid pδGAPb digested by *Nde* I and *Nhe* I, generating plasmid pδGAPb-PDI.

Then, 5-10 μg of each constructed plasmid was linearized by digestion with restriction enzyme *Not* I and transformed into *S. cerevisiae* using the lithium acetate method. The positive transformants were selected on YPD plates with addition of the antibiotic geneticin for pδGAPg-CYS4 (G418, 4 mg/ml), hygromycin B for pδGAPh-CYS3 (HgyB, 1 mg/ml), or blasticidin S for pδGAPb-PDI (Bla, 500 μg/ml), and verified by DNA sequencing after the integrated gene fragments were amplified by PCR using the corresponding primers and genomic DNA templates.

Deletion of *STR3* in *S. cerevisiae*

To disrupt *STR3* gene, a homologous integration fragment with a selective marker, zeocin resistance gene, was amplified by PCR from the plasmid pδGAPz. The primers STR3_H1 and STR3_H2 (Supplementary Table S8) were used for the amplification of homologous fragment of the *STR3* gene. The resulting DNA fragment was purified

using the EasyPure PCR Purification Kit (TransGen, Beijing, China). Then, 5-10 μg of the purified fragments were transformed into *S. cerevisiae* using the lithium acetate method. Transformants were screened on YPD plates with addition of antibiotic zeocin (750 μg/ml) and verified by PCR using the corresponding primers and genomic DNA as a template. The amplified DNA fragments of the positive constructs were further verified by DNA sequencing (Taihe, Beijing, China).

CRISPR/Cas9-mediated reverse mutation assay

The commercially available expression vectors used for *S. cerevisiae* metabolic engineering almost carry a nutritional marker for selection in yeast. These selection markers hamper a reverse mutation to prototrophy in this work. Thus, a derivative (pGAD-Cas9-Bla) of plasmid pGADT7 (clontech) with a blasticidin resistance gene was constructed as follows: the 929-bp phosphoglycerate kinase 1 (PGK1) promoter was generated by PCR amplification using primers PGK1_Sph and PGK1_Nde (Supplementary Table S9) and yeast genome DNA as a template. The PCR fragment was digested with *Sph* I and *Nde* I, gel-purified, and then ligated into plasmid pAGDT7, which was digested with the same enzyme pair. The final plasmid was designated as pGAD-PGK1. The DNA fragments of Cas9 gene, a codon-optimized version originally constructed for expression in human cells[1], were amplified using the primer pairs X260_ 1/X260_2 and X260_ 3/X260_4 (Supplementary Table S9), and inserted step by step into plasmid pGAD-PGK1, generating plasmid pAGD-Cas9. Next, the *LEU2* selection marker was replaced by an amplified DNA fragment using primers GADT7_H3 and GADT7_Nhe and plasmid pGADT7 as a template. At last, a

DNA fragment containing an ORF of antibiotic selection marker, a blasticidin resistance gene, was obtained by PCR amplification using the primers Bla_Nhe and Bla_Not and plasmid pPIC6/*lacZ* (Invitrogen life technologies) as a template, sub-cloned into the intermediate plamid pGAD-Cas9-Leu$^-$ to generate the final plasmid pGAD-Cas9-Bla.

gRNA expression Cassettes (containing the snoRNA *SNR52* promoter, guide RNA structre sequence and *SUP4* terminator) were constructed following the method as previously reported[2]. Firstly, a DNA fragment of snoRNA *SNR52* promoter with a guide gRNA seqence for *ADE2* gene was obtained by SOE-PCR with primers from SNR_G1 to SNR_G7 (Supplementary Table S9), further amplifying with primers SNR_G1 and ADE_G1 to get a recombinant DNA fragment *SNR52-gRNA*$_{ADE2}$ (295 bp). Primers SNR_G8 and SNR_G9 (Supplementary Table S9) were used to run a complementary reaction by PCR. A second PCR amplification was run using the primers ADE_G1 and SNR_G9 to generate a recombinant DNA fragment *gRNA*$_{ADE2}$*-SUP4* (120 bp) with gRNA structure. Next, a further complementary reaction between 295 bp and 120 bp fragments was carried out. At last, an expression Cassette of gRNA for *ADE2-1* allele gene (the expected length 395 bp) was amplified by SOE-PCR with primers SNR_G1 and SNR_G9 from the product of complementary reaction. Fragment *SNR52-gRNA*$_{ADE2}$*-SUP4* was cloned into a T-vector EZ-T (a derivative of pBluescript II KS(+), GenStar, Beijing, China) via TA cloning to give EZ-gRNA-Ade and verified by DNA sequencing. Other expression Cassettes of gRNAs for *URA3-1*, *LEU2-3*, *TRP1-1* and *HIS3-11* allele genes were

amplified using two sets of primer pairs Ura_G1/M13$^+$, Leu_G1/M13$^+$, Trp_G1/M13$^+$, His_G1/M13$^+$ and Ura_G2/M13$^-$, Leu_G2/M13$^-$, Trp_G2/M13$^-$, His_G2/M13$^-$ (Supplementary Table S9), respectively, and plasmid EZ-gRNA-Ade as a template. The corresponding two DNA fragments purified was further carried out a complementary PCR reaction. Then, the corresponding PCR products as templates and a same primer pair SNR_G1/SNR_G9 were used to amplify the gRNA expression Cassettes which express to synthesize the gRNAs for *URA3-1*, *LEU2-3*, *TRP1-1* and *HIS3-11* allele genes.

Donor DNAs corresponding to *ADE2*, *URA3*, *LEU2*, *TRP1* and *HIS3* genes were directly amplified using the primer pairs Ade1/Ade2, Ura1/Ura2, Leu1/Leu2, Trp1/Trp2 and His1/His2, respectively, and the wild yeast genomic DNA as a template. The strain expressing Cas9 was constructed by transforming the Cas9 expression plasmid pGAD-Cas9-bla using the LiAc/SS carrier DNA/PEG method. The gRNA cassettes and donor double-stranded oligonucleotides were co-transformed into W303-1b/FGP/pGAD-Cas9-bla cells as follows: A colony was inoculated in 10 ml liquid YPD media supplemented with 75 μg/ml of blasticidin and grown overnight. The next morning, a 1-ml culture was transferred to 20 ml liquid YPD media and incubated for additional 5-6 h until OD_{600} of 0.8 to 1.0. Cells were collected via centrifugation at 4000 rpm for 5 min and the media was removed. The cell pellet was washed once by 10 ml ice-cold water and once again by 10 ml of ice-cold 100 mM LiAc. The cells were conditioned by re-suspending and co-transformed 1μg of each gRNA cassette, 5 μg of each donor DNA and 50 μg of salmon sperm carrier DNA.

The reverse mutation assay was divided into three stages to conduct. In the first stage, yeast transformation was carried out to transform the $gRNA_{ADE2}$ and $gRNA_{URA3}$ cassettes and the donor DNAs of *ADE2* and *URA3* genes. The positive clones were screened on the synthetic complete (SC) plates supplemented with 75 μg/ml of blasticidin and without adenine and uracil, and verified by DNA sequencing after the gene fragments were amplified by PCR using the corresponding primers and the genomic DNA as a template. Then, the prototrophic mutant of *LEU2-3* and *TRP1-1* allele genes was obtained by co-transformation of the $gRNA_{LEU2}$ and $gRNA_{TRP1}$ cassettes and the donor DNAs of *LEU2* and *TRP1* genes. At last, the W303-1b/FGP^{PT} strain was generated by transformation of the $gRNA_{HIS3}$ cassette and the donor DNA of *HIS3* gene.

Growth behaviors of prototrophic strain in YPD medium

The growth behaviors of different strains were analyzed in YPD medium. Single colonies on YPD plates were inoculated into a 100-ml conical flask with 10 ml medium. The cultures were incubated at 30 ℃ and 220 rpm for 24 h and subsequently inoculated into a 250-ml conical flask containing 50 ml medium with an initial OD_{600} of 0.2, which was then incubated at 30 ℃ and 220 rpm. Samples were taken out at different growth stages for the detection of DCW and GSH production.

Determination of biomass and stability analysis of GSH production

The DCW was determined gravimetrically and showed a functional relationship with the spectrophotometric measurement of turbidity at 600 nm (OD_{600}). A 5-ml sample

was harvested, and the cells were washed twice by distilled water and dried at 100 °C for 24 h. OD_{600}, GSH extraction and determination were performed as previously reported[3].

The stability of the GSH production was analyzed in WMVIII medium. The cells of all strains were inoculated in WMVIII medium with an initial OD_{600} of 0.5. The cells were then transferred into fresh medium when they grew to the mid-exponential growth phase (OD_{600} = 15-30) until 100 generations. An aliquot of the yeast cultures was removed and stored for the determination of the DCW and GSH production.

Strain mutagenesis and adaptive evolutionary experiments

The strain mutagenesis of the engineered strain W303-1b/FGP^{PT} was pretreated using 50 μg/ml of N-methyl-N'-nitro-N-nitrosoguanidine (MNNG), and the killing rate was ~90%. The MNNG-treated strain was designated as W303-1b/FGP^{PT}m. The ALE experiments were carried out as previously reported[4], but with a slight modification. Briefly, cells of W303-1b/FGP^{PT} and W303-1b/FGP^{PT}m were both inoculated in WMVIII medium with an initial OD_{600} of 0.5. The cells were transferred into fresh medium when they grew to the mid-exponential growth phase (OD_{600} = 15-30) with an increasing concentration of Acr and a low level of 5 μg/ml MNNG or not. The single clone analysis of GSH production was carried out after approximately 100 generations.

In the second round of adaptive evolution, the selected isolate W303-1b/FGP^{PT}-8 was grown in WMVIII medium. the initial concentration of Acr was 0.42 mM and no additional MNNG was added.

Effects of Acr on the growth behavior of *S. cerevisiae* cells

To test the initial concentration of Acr in the ALE experiments, yeast cells were incubated in WMVIII medium with an initial OD_{600} of 0.2 under the condition of different concentrations of Acr. The cultures were incubated at 30 ℃ and 220 rpm. OD_{600} was measured at 48 h and 96 h (Supplementary Table S1).

To evaluate the effects of Acr on the survival rate of the evolved strain W303-1b/FGP^{PT}-8-12, yeast cells were incubated in WMVIII medium and grown to the mid-exponential growth phase (OD_{600} = 15-30). The cultured cells diluted to an optical density at 600 nm (OD_{600}) of 0.5 were exposed to Acr for 2 h at 30 ℃ in WMVIII medium. After centrifugation, Cell pellets were washed twice with fresh WMVIII medium and plated onto WMVIII plates following serial dilution. Number of colonies per plate were counted and compared after the plates were incubated at 30 ℃ for three days (Supplementary Table S2).

Extraction of metabolites and Metabolomic analysis

Both W303-1b/FGP^{PT} and W303-1b/FGP^{PT}-8-12 strains were grown in WMVIII medium and incubated at 30 ℃ and 220 rpm for regular intervals of 12, 24, 36, 48 and 60 h. 30 ml of each culture was removed and rapidly quenched by cooling down the mixture on ice in 50-ml Falcon tubes. Then, all tubes were centrifuged at 8,000 rpm for 15 min at 4 ℃. The supernatant was discarded, and yeast cell pellets in the tube were washed with PBS buffer. 25 ml of PBS buffer was poured into each sample, the Falcon tubes were centrifuged at 8,000 rpm for 10 min at 4 ℃, and the supernatant was removed. This step was repeated twice.

The yeast metabolites were extracted using boiling ethanol (BE)[5]. 5 ml of 75% (v/v) ethanol that had been preheated in a water bath at 95 °C for 5 min was added to the cell pellets, and the suspension was further incubated for 3 min at 95 °C. After cooling down the mixture on ice, all the samples were centrifuged at 12,000 rpm for 5 min at 4 °C. The supernatant was taken out, concentrated and dried by evaporation using nitrogen gas. The residue was then resuspended to a final volume of 0.5 ml with Milli-Q water.

The metabolic samples were analyzed by relatively quantitative measures (i.e., fold change across biological conditions) using an internal standard method. Prior to the LC-MS/MS analysis, 100 μL of the metabolites was centrifuged at 8,000 rpm for 15 min at 4 °C and then 20 μL of 0.5 mM 2-isopropylmalic acid was added as an internal standard. 200 μL of acetonitrile was added to remove proteins from the metabolites. The mixture was centrifuged at 12,000 rpm for 10 min, and the supernatant was collected for LC-MS/MS.

The mixture was analyzed using the "cell culture profiling" package on LC-MS/MS system (Shimadzu, Japan), which makes possible the simultaneous analysis of 95 components at a rate of 17 minutes per sample. The 95 components consist of a wide variety of metabolites: 5 sugars, 38 amino acids, 17 vitamins, 18 nucleic acid associated substances and 17 others. Instrumental analysis was carried out as follows: UHPLC analysis was performed on a UHPLC 30AD system (Shimadzu, Japan). Analyst LabSolutions LCMS version 5.82 software and an 8060 triple-quadrupole mass spectrometer with an electrospray ionization (ESI) source

were used for the master control of LC and MS recorder following operation parameters: capillary voltage 4000 V, collision gas pressure 270 kPa, DL temperature 250 °C, interface temperature 300 °C, heat block temperature 400 °C, nebulizing gas flow 3 L/min, heating gas flow 10 L/min and drying gas flow 10 L/min.

To convert raw LC-MS/MS spectral data to relative cellular concentration data, the spectral data were normalized as follows:

$$\text{Normalized spectral data} = \text{Raw spectral data} \times (\text{Raw spectral data}_{\text{internal standard}})^{-1}. \quad (1)$$

The relative concentrations were calculated by dividing normalized spectral data by the corresponding value of DCW:

$$\text{Relative concentration} = \text{Normalized spectral data} \times (\text{DCW})^{-1} \quad (2)$$

The fold change of a special metabolite was calculated as follows:

$$\text{Fold change} = \text{Relative concentration}_{\text{evolved strain}} \times (\text{Relative concentration}_{\text{parental strain}})^{-1}. \quad (3)$$

Fold changes were used to evaluate the specific metabolite in the evolved strain W303-1b/FGP^{PT}-8-12 and the parental strain W303-1b/FGP^{PT}. Three situations might occur: If the change was >1, it meant that the concentration of the specific metabolite in the evolved strain was higher than that in the original strain while if the ratio was =1 or <1, it meant that the concentration of the specific metabolite was equal to or lower than that in the basic strain (Supplementary Table S3).

Identification of Acr-thiol adducts and Determination of Acr-GSH *in vivo*

The parental strain W303-1b/FGP^{PT} were incubated in WMVIII medium for 24 h and diluted to OD_{600} of 0.5 in 100 ml of fresh WMVIII medium. Then 1.8 mM Acr was

added. This mixture was centrifuged at 8,000 rpm for 15 min at 4 ℃ following incubated at 30 ℃ and 220 rpm for regular time intervals. The supernatant was stored at -80 ℃ for further use and the yeast pellets were washed with PBS and extracted three times using BE. Then both the supernatant and the yeast extract were analyzed by LC-MS/MS.

Prepared Acr-GSH was used as the standard to monitor accumulation kinetics of Acr-GSH in vivo using LC-MS/MS. Six standard solutions of Acr-GSH was prepared by a serial dilution from 1 mM to 1×10^{-5} mM. 100 μL of Acr-GSH solution was centrifuged at 8,000 rpm for 15 min at 4 ℃ and then 20 μL of 0.5 mM 2-isopropylmalic acid was added as an internal reference standard. 200 μL of acetonitrile was added to remove proteins. The mixture was centrifuged at 12,000 rpm for 10 min, and the supernatant was collected for LC-MS/MS analysis. The correction factor of Acr was calculated through the following equation:

$$f= (A_i\times m_i^{-1}) \times (A_s\times m_s^{-1})^{-1} \quad (4)$$

A_i and A_s are peak area of the internal standard and the standard solutions of Acr-GSH. m_i and m_s are the concentration of internal standard and the standard solutions. The calculated correction factor was 8.99.

The concentration of Acr-GSH was calculated as followings:

$$m_q = f\times A_q\times(A_i\times m_i^{-1})^{-1} \quad (5)$$

m_q and A_q are peak area and concentration of Acr-GSH in the quenched metabolites.

The diffusion rate of Acr into yeast cells is calculated by the equation: $R=N\cdot min^{-1}\cdot OD_{600}^{-1}$. N is the accumulated Acr-GSH adduct. And the total OD_{600} of the

treated cells was 50.

Synthesis of Acr-thiol adducts

4.5 μmol Acr (MW 56) was added to a stirring suspension of L-Cys (MW 121) (1.2 mg, 10 μmol) in 500 ml of water, and the mixture was stirred overnight at 30 °C. The products were monitored by an LC-MS-8060 triple quadrupole mass spectrometer in multiple reaction monitoring (MRM) mode. The calculated mass for the Acr-Cys adduct $C_9H_{16}N_2O_4S_2$ was 280. ESI mass spectrometry (MS) showed a quasi-molecular ion (M - H^+) at m/z 279.15 and (M + H^+) at m/z 281.05. The Acr-homocysteine (Acr-Hcy) and Arc- *N*-acetylcysteine (Arc-NAC) adducts were all synthesized following the procedure of Acr-Cys synthesis. The resulting adducts Acr-Hcy and Acr-NAC were confirmed by ESI-MS.

Acr-GSH was obtained by incubating GSH (40 mM) in the presence of 60 mM Acr in 1 mM PBS for 24 h at 30 °C. Sample aliquots were directly separated by HPLC for the purification of Acr-GSH (MW = 363). The collected fractions were concentrated by lyophilization overnight to give Acr-GSH, a white crystal powder. Samples were partially dissolved in H_2O:CH_3OH 90:10 (v/v) and filtered through 0.45-μm nylon filters before analyzed by ESI-MS (direct injection) as described above. Another samples were dissolved in D_2O and tested by NMR. The ^1H and ^{13}C chemical shift assignments were showed in Supplementary Table S4.

Results

Construction of CRISPR/Cas9 gene editing system and yeast transformation. CRISPR/Cas9 is a simple and efficient tool for targeted and marker-free genome

engineering. Though the multiplex CRISPR/Cas9 systems for genome engineering of up to 5 different genomic loci in one transformation step in baker's yeast have been developed, not only they depend on single or dual plasmids for expressing Cas9 and gRNA(s)[6,7], multiplex constructions of gRNA expression cassettes but also are time-consuming. In addition, these plasmid vectors usually with a nutritional marker, such as *LEU2* and *URA3*, or even more, used for selection in yeast, impede this reverse mutation assay. Therefore, we had to construct an episomal plasmid pGAD-Cas9-bla with an antibiotic selection marker, a blasticidin resistance gene, conveniently to select the prototrophic reversion mutant (Supplementary Fig. S13).

The parental *S. cerevisiae* strain, W303-1b/FGP, has five auxotrophic alleles and does not grow in the mineral salt medium. To reversely mutate this strain and shorten the time needed for efficient gene editing, we attempted to employ a transient co-transformation of two gRNA PCR cassettes (e.g., $gRNA_{ADE2}$ and $gRNA_{URA3}$) along with the corresponding two linear donor DNAs of *ADE2* and *URA3* genes, as reported that co-transformation of the CRISPR−Cas9 system and a linearized HR donor DNA in *S. cerevisiae* had been carried out by others[2]. In this positive selection, *ADE2-1* and *URA3-1* alleles were corrected through a single transformation event, while mutations causing correction of the auxotrophic mutation would be rare without donor DNA. Additional two rounds of transient co-transformation were performed to correct the other three *LEU2-3*, *TRP1-1* and *HIS3-11* alleles with positive selection marker for each assay, generating the prototrophic strain W303-1b/FGP^{PT}. But, we do not compare the gene editing efficiency of triple, quadruple and quintuple

combinations. The final prototrophic strain W303-1b/FGP^{PT} was verified by DNA sequencing (Supplementary Fig. S14) after the gene fragments were amplified by PCR using the corresponding primers and the genomic DNA as a template.

GSH production of the auxotrophic and prototrophic strains. The GSH production and biomass of strains W303-1b/FGP and W303-1b/FGP^{PT} were measured at given time intervals. Both engineering strains reached a maximum DCW of approximately 9.3 g/L and a highest GSH production of approximately 216 mg/L. However, it took the auxotrophic strain 96 h to reach the stationary phase, while the prototrophic W303-1b/FGP^{PT} grew to the stationary phase at 48 h (Supplementary Fig. S1).

References

1. Cong, L. *et al.* Multiplex genome engineering using CRISPR/Cas systems. *Science* **339**, 819-823 (2013).

2. DiCarlo, J. E. *et al.* Genome engineering in *Saccharomyces cerevisiae* using CRISPR-Cas systems. *Nucleic acids research* **41**, 4336-4343 (2013).

3. Tang, L. *et al.* Three-pathway combination for glutathione biosynthesis in *Saccharomyces cerevisiae*. *Microbial cell factories* **14**, 139 (2015).

4. Patzschke, A. *et al.* Enhanced glutathione production by evolutionary engineering of *Saccharomyces cerevisiae* strains. *Biotechnology journal* **10**, 1719-1726 (2015).

5. Canelas, A. B. *et al.* Quantitative evaluation of intracellular metabolite extraction techniques for yeast metabolomics. *Analytical chemistry* **81**, 7379-7389 (2009).

6. Jakočiūnas, T. *et al.* Multiplex metabolic pathway engineering using CRISPR/Cas9

in *Saccharomyces cerevisiae*. *Metabolic engineering* **28**, 213-222 (2015).

7. Bao, Z. *et al.* Homology-integrated CRISPR–Cas (HI-CRISPR) system for one-step multigene disruption in *Saccharomyces cerevisiae*. *ACS synthetic biology* **4**, 585-594 (2014).
